# Do fish gut microbiotas vary across spatial scales? A case study of *Diplodus vulgaris* in the Mediterranean Sea

**DOI:** 10.1186/s42523-024-00319-2

**Published:** 2024-06-13

**Authors:** Ginevra Lilli, Charlotte Sirot, Hayley Campbell, Fanny Hermand, Deirdre Brophy, Jean-François Flot, Conor T. Graham, Isabelle F. George

**Affiliations:** 1 Laboratoire d’Ecologie des Systèmes Aquatiques (ESA), Université Libre de Bruxelles (ULB), 1050 Brussels, Belgium; 2 Centre de Recherches Insulaires et Observatoire de l’Environnement (CRIOBE), University of Perpignan, Perpignan, France; 3 Marine and Freshwater Research Centre, Atlantic Technological University, Dublin Road, Galway, Ireland; 4 Evolutionary Biology and Ecology, Université libre de Bruxelles (ULB), 1050 Brussels, Belgium; 5 Interuniversity Institute of Bioinformatics in Brussels – (IB)², 1050 Brussels, Belgium

**Keywords:** Fish gut microbiome, Mediterranean Sea, Sparidae, *Posidonia oceanica*, Biogeography, Spatial variation, Benthic habitat

## Abstract

**Background:**

Biogeography has been linked to differences in gut microbiota in several animals. However, the existence of such a relationship in fish is not clear yet. So far, it seems to depend on the fish species studied. However, most studies of fish gut microbiotas are based on single populations. In this study, we investigated the gut microbiota of fish from three wild populations of the two-banded sea bream *Diplodus vulgaris* (Geoffroy Saint-Hilaire, 1817) to determine whether its diversity, structure and potential functionality reflect the geographic origin of the fish, at large and small geographical scale. Additionally, we explored the host- and environmental-related factors explaining this relationship.

**Results:**

We showed that the taxonomy and potential functionality of the mucosa-associated gut microbiota of *Diplodus vulgaris* differ to varying degrees depending on the spatial scale considered. At large scale, we observed that both the taxonomical structure and the potential functionality of the fish microbiota differed significantly between populations. In contrast, the taxonomical diversity of the microbial community displayed a significant relationship with factors other than the geographic origin of the fish (i.e. sampling date). On the other hand, at small scale, the different composition and diversity of the microbiota differ according to the characteristics of the habitat occupied by the fish. Specifically, we identified the presence of *Posidonia oceanica* in the benthic habitat as predictor of both the microbiota composition and diversity. Lastly, we reported the enrichment of functions related to the metabolism of xenobiotics (i.e. drugs and 4-aminobenzoate) in a population and we indicated it as a potential target of future monitoring.

**Conclusions:**

With this study, we confirmed the importance of investigating the gut microbiota of wild fish species using multiple populations, taking into account the different habitats occupied by the individuals. Furthermore, we underscored the use of the biodegradation potential of the gut microbiota as an alternative means of monitoring emerging contaminants in Mediterranean fish.

**Supplementary Information:**

The online version contains supplementary material available at 10.1186/s42523-024-00319-2.

## Background

In the sea, more than 90% of the total biomass is represented by microbial cells [1]. A portion of these microbial organisms are fish pathogens, but others have established mutualistic relationships with fishes [2–4]. The communities of microorganisms residing in the intestinal system are known as gut microbiota [5, 6] and their contribution to the immune response and nutrition has been largely recognized in fishes [7]. This community can prevent the invasion of pathogens both by competing with those for space, nutrients and adhesion receptors and by modulating an inflammatory response through the production of short chain fatty acids (SCFAs) such as acetate, propionate and butyrate [7]. Furthermore, through the biosynthesis of enzymes such as chitinases, cellulases, proteases and lipases, it supports the host in the catabolism of otherwise indigestible diet components [7].

So far, studies on the relationship between fish hosts and gut microbes have mostly focused on fishes raised in controlled environments such as in aquaculture facilities or laboratories [8]. These studies have been paramount in advancing our understanding of key physiological aspects of gut microbiota, such as its impact on the gut-brain-axis [9] or in the metabolism of specific nutrients [10], and to optimize both rearing conditions in aquaculture and the nutritional value of the fishes [11]. Yet, it is likewise crucial to extend investigation of gut microbiota to teleost species living in the wild, particularly when one considers that wild fishes still represent half of the total fishes consumed in the world (even 74% in Europe) [12]. In fact, monitoring the status of the gut microbiota of wild fishes can provide—when combined with conventional body condition indices [13]—crucial information regarding the health status of the fish populations [14, 15] and support the assessment and management of fish stocks [16]. For example, fish populations with less diverse microbiota (i.e. microbiota with low values of alpha diversity) may be more susceptible to pathogens and parasite colonization [17]. In addition, the absence of key bacterial genera in the microbiota of a fish species may alter its metabolism and homeostasis, as was reported for the genus *Mycoplasma* in the Atlantic salmon (*Salmo salar* L.) [18]. Finally, a fish population experiencing acute stress may exhibit greater inter-individual dissimilarity of the composition of the gut microbial community [15, 19].

So far, most of the studies investigating wild fishes have included specimens from single geographic locations [20–22]. Nevertheless, biogeography of the host has been linked to differences in the gut microbiota across the animal kingdom, from corals to humans passing through birds and wild rodents just to cite a few [23–26]. In fishes, the relationship between the host’s geographic origin and the features of the gut microbiota has not been completely unraveled yet. A recent study including individuals from 24 species sampled along the Yellow river in China (1500 km) has shed some light on the effect of biogeography on the fish gut microbiota [27]. This study reported that both the gut microbiota composition and diversity differed depending on the sampling location selected along the river; furthermore, it showed that the larger the distance between the sampling locations, the greater the dissimilarity in the composition of the fish gut microbiota. Similarly, the composition of the gut microbiota was observed to differ depending on the geographic origin of the host for two species of *Siganus* sp. sampled in the Red Sea and in the Mediterranean Sea [28], and three species of *Scorpaena* sp. sampled in different regions of the Mediterranean Sea [29]. Differently, geographical origin did not significantly explain the dissimilarities observed in the gut microbiota of sea bream (*Sparus aurata*) and sea bass (*Dicentrarchus labrax*) farmed in two geographically distant locations in Greece [30], or in that of Atlantic salmon (*Salmo salar*) living in the wild in Ireland and Canada [31]. Therefore, the presence of a relationship between the geographic origin of the marine fishes and the composition of their gut microbiota seems to depend on the fish species investigated. Extending the analysis of the gut microbiota to a large geographical range could also contribute to depict better the core microbiota of a wild fish species. The core microbiota is described as the set of microbial taxa that, by being shared by the majority of the individuals, characterizes a species [32]. Fish populations with bacterial communities greatly divergent from the core could then be investigated to determine the causes and the consequences of such departure. A similar approach has been undertaken for humpback whales (*Megaptera novaeangliae*) by characterizing the skin microbiota of different populations, which assisted in the description of a ‘natural’ healthy microbiota that could be used as baseline for conservational studies [33].

Both host- and environmental-related factors may underlie the variation in fish gut microbiota across a geographical range. Among the former, the genetic divergence between the individuals of different populations may be reflected in a greater dissimilarity in the composition of their gut microbiota, as it was observed for ten Canadian populations of threespine stickleback (*Gasterosteus aculeatus*) from different locations [34]. In contrast, while the trophic level occupied by a species has been useful to understand the differences in the gut microbiota structure and diversity across fish species [2, 7], it is less likely to be helpful in unravelling their variation through a geographical range in individuals of the same species.

Among the environment related variables, the nutrient load in the water column, the resources available for the fishes and the composition of the backterioplankton communities ingested by fish during osmoregulation and, in some fish species, for feeding, are described to directly influence the structure of the gut microbiota in fishes [2, 7, 35, 36]. Water temperature influences the two former variables [7], and together with the salinity can affect the latter [35]. The diet composition driven by the availability of resources in the different geographic locations might determine changes in the composition of the bacterial community [8]: for example, the presence of insects in the diet was found to result in an increase in the abundance of chitin-degrading bacteria in the Atlantic salmon, while increased concentration of fatty acids in the diet was correlated to greater microbial diversity in juvenile golden pompano (*Trachinotus ovatus*) and to greater abundance of *Pseudomonas* in zebrafish (*Danio rerio*) [37]. However, a more diverse diet does not necessarily imply a more diverse gut microbiota in fishes: for example, specialist individuals of threespine stickleback and Eurasian perch (*Perca fluviatilis*) were observed to display a more diverse microbial community than their generalist relatives [38].

The habitat occupied by fishes has also been observed to influence the structure of their gut microbiota: the microbiota of fishes from freshwaters and marine environments was extensively demonstrated to be taxonomically different [39–41]. In contrast, the influence of the type of benthic habitats (e.g. rocky reef, soft bottom, seagrass meadows etc.) on the gut microbiota of wild fishes has been little investigated [2, 19, 42]. In [42], Minich et al. observed a significant relationship between the type of benthic substratum and the microbial biomass, diversity and composition of the fish gut microbiota. This was described as a consequence of the influence of the benthic substratum on the composition of the backterioplankton community, which would in turn affect the microbial community associated with the fishes [42].

Finally, across a geographical range, the habitat of a fish species can be characterized by different levels of human pressure. The influence of habitat degradation on the gut microbiota of fish has been sporadically investigated to date [19, 43, 44], and no investigation has been conducted to explore the influence of habitat protection on the microbiota associated with fish body niches (i.e. gills, skin and gut). The protection of the marine habitat through Marine Protected Areas (MPAs) and more extensively through Fully Protected marine Areas (FPAs) (i.e. areas of full protection where any human activity is forbidden) is recognized to support an increase in abundance, biomass, diversity, body size and reproductive output of the macro-organisms species living within them [45, 46] and in their surroundings [47]. Furthermore, sediment samples collected within marine protected areas were reported to harbor a more diverse microbial community than those sampled in areas undergoing different types of anthropogenic disturbance [48, 49]. Finally, habitat protection has already been observed to be a predictor of microbial communities associated with terrestrial animals [50, 51]. In light of this, a possible influence of habitat protection on the gut microbiota of fishes cannot be excluded and further investigation should be performed to explore such a relationship.

The common two-banded sea bream *Diplodus vulgaris* (Geoffroy Saint-Hilaire, 1817) is an ecologically important demersal fish species mainly found in habitats with rocky and sandy-muddy substrates and seagrass beds down to a depth of 160 m but it is mostly recorded at depths of 30 m and less. Its distribution range extends from the eastern Atlantic ocean (from the Bay of Biscay to Senegal) to the Black Sea and the Mediterranean Sea [52]. In the latter region, it represents a commercially valuable resource for local fisheries, as it accounts for 14% and 5% of the total weight of demersal and pelagic gillnets catches, respectively [53]. It is a generalist omnivore mainly relying on crustaceans, mollusks, polychaetes and small fishes [54, 55]. As reported in [56], *D. vulgaris* is an opportunistic feeder, exploiting both small to large preys according to its size and to the prey availability in the environment. Furthermore, both in the Mediterranean Sea and in the Atlantic Ocean, *D. vulgaris* was observed to display small home ranges and high site fidelity (i.e. the tendency to return to a previously occupied location) [57, 58]. The generalist feeding behavior and the small home range of this fish makes it an ideal model species to study the effect of geographic location and habitat features on the gut microbiota for two main reasons. First, this species is able to acclimatize and thrive in different environments and to exploit the resources available [59]: this is expected to result in changes in the composition of the gut microbiota without major losses in the overall microbial diversity, as already observed for other species of generalist fish [38]. Secondly, the small home range typically displayed by *D. vulgaris* facilitates the study of the effect of local environmental variables even over small spatial scales.

This study examines the gut mucosa microbiota of *D. vulgaris* inhabiting three distant regions of the French Mediterranean coast. The primary aim was to identify the bacterial taxa that occur in the majority of the individuals from the three regions and therefore to characterize the core gut microbiota of this wild species. Spatial variability in the taxonomical structure and diversity and the potential functionality of the gut microbiota of *D. vulgaris* was evaluated both at a large (> 100 km between sites) and at a small spatial scale (< 100 km between the sites of one single region). Lastly, the role of the benthic habitat displayed in the home range of *D. vulgaris*, the distance from the FPAs and the diet of the individuals was investigated to determine their contribution to the spatial variation of the gut microbiota structure and functionality at a local scale.

## Materials and methods

### Sampling collection

**Fig. 1:**
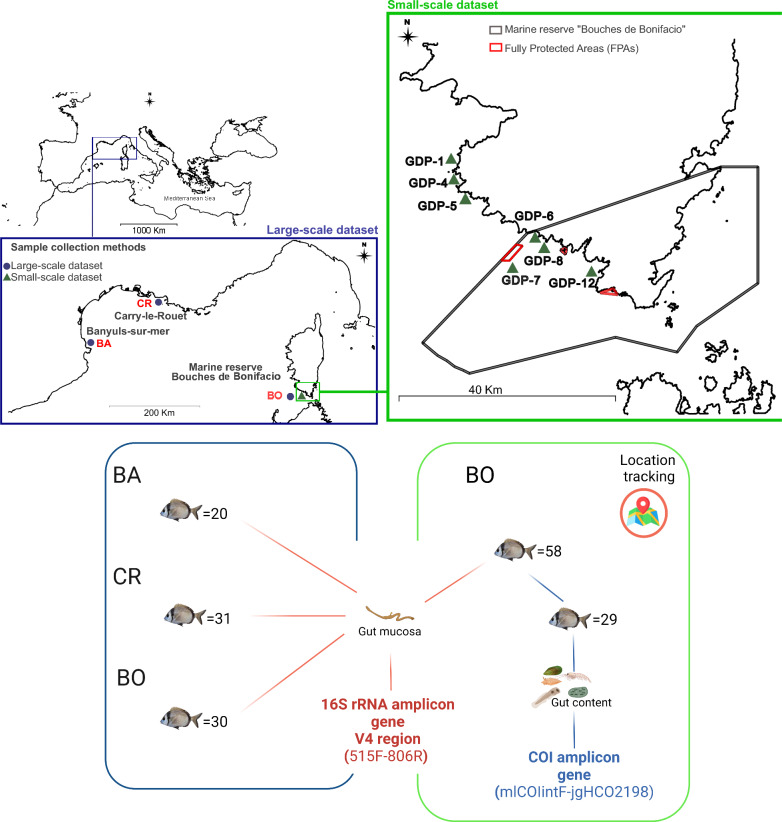
Summary of the design of the study. (i) The geographic locations where the sampling of *D. vulgaris* took place, (ii) the number of samples collected in each location and included in each dataset. GDP: Geographic Distance Point and (iii) the gene markers targeted in this study

Samples were collected in three regions of the French Mediterranean coast in the North-Western Mediterranean Sea (Fig. 1). Two of these regions are located along the coast of the Lion Gulf: the first one is in close proximity to the marine reserve of Cerbère-Banyuls and the second one includes the portion of coastal sea between the marine reserves of Couronne and Carry-le-Rouet. The third region is along the south-western coast of the French island of Corsica and partially included in the marine reserve of Bonifacio. For the sake of simplicity, the three regions will be referred to as respectively “BA”, “CR” and “BO”. The three regions differ in terms of marine habitat types: BA is characterized by a coralligenous habitat, CR by *Posidonia oceanica* meadows extending up to 1 km of distance from the coast and BO by patches of *Posidonia oceanica* meadows alternating with detritic, rocky and sandy bottoms (EUSeaMap 2021—EMODnet broad-scale seabed habitat map for Europe, Mickaël Vasquez, 2021). Furthermore, the BO region is characterized by higher water temperature throughout the year compared to BA and CR (MARS3D model simulations, [60]). Lastly, according to the maps available on www.medtrix.fr in the IMPACT project [61], the level of cumulative anthropogenic impact in the three regions is similar. However, the sources of this impact differ across them: the land adjacent to BA and CR is more urbanized than BO; CR is more subjected to the presence of industrial effluents than the two other regions, due to its proximity to the industrial site of Fos-Barre, while coastal agriculture is still an important source of disturbance both in BA and in BO.

In total, 81 *D. vulgaris* specimens were collected between the 29th of July and the 1st of September 2021 by professional fishermen in the three regions by using gillnets and longlines (20 individuals in BA, 31 in CR and 30 in BO) (Fig. 1). The specimens caught by the fishermen were collected by us once already dead, directly at the dock. Because the three regions are more than 100 km away from each other—specifically, BA and CR are separated by 180 km while BO is more than 350 km away both from BA and from CR—the individuals collected in these regions were included in the analyses to define the effect of geographical location on the gut microbiota taxonomy and potential functionality at a large spatial scale. This dataset will be referred to as “large-scale dataset” (n = 81). Information about the water temperature on the sampling days in the three regions was retrieved from [60].

Fifty-eight additional fish were collected by us in BO between the 30th of August and the 2nd of September 2021 at different distances from fully protected areas (FPAs) through line-fishing with the help of professional fishermen (Fig. 1). Specimens collected in this way were immediately euthanized by cervical dislocation (following the European directive 2010/63/UE). The exact coordinates of the sampling locations were recorded for these specimens (GPS points, Supplementary Metadata). The maximum distance between the sampling locations included in this additional sampling was equal to 33.6 km, therefore these samples were employed to study the effect of geographic location at a small spatial scale and the dataset will be referred to as “small-scale dataset” (n = 58). No sample was collected directly from inside the fully protected areas.

The total length of the fish (i.e. from the tip of the snout to the tip of the longer lobe of the caudal fin) was recorded. Fish were kept on ice immediately post capture and dissected within a maximum of three hours from their collection. The skin was rubbed with a sterile tissue and by adding ethanol 70% prior to dissection to avoid the contamination of the samples by the skin microbiota. Then the middle and posterior portions of the intestine (midgut and hindgut) of each individual were extracted. The intestinal content (digesta) was collected in 50-ml tubes and stored in 96% ethanol for diet analyses. Although intestines were collected for all the fish, only the diet of a subset of the small-scale dataset (N = 29) was analyzed (Fig. 1; Supplementary Metadata).

Once the intestine was emptied of digested food, the intestinal wall was rinsed with PBS 1× to remove any food remains and to keep only the bacterial community adherent to it (i.e. autochthonous community). Intestinal wall samples were placed in sterile 50-ml collection tubes and completely immersed in up to five times their volume of RNAlater (Sigma-Aldrich). Following an overnight incubation at 4°C, they were stored together with the diet samples at − 20°C for the rest of the field campaign (~ 55 days maximum) and eventually at − 80°C until DNA extraction (in November 2021).

### Characterization of the habitat features of the home ranges of *D. vulgaris* and measurement of distance from fully protected areas

The benthic habitat type and the distance from the fully protected areas were investigated only for the individuals included in the small-scale dataset (Supplementary Metadata).

**Fig. 2:**
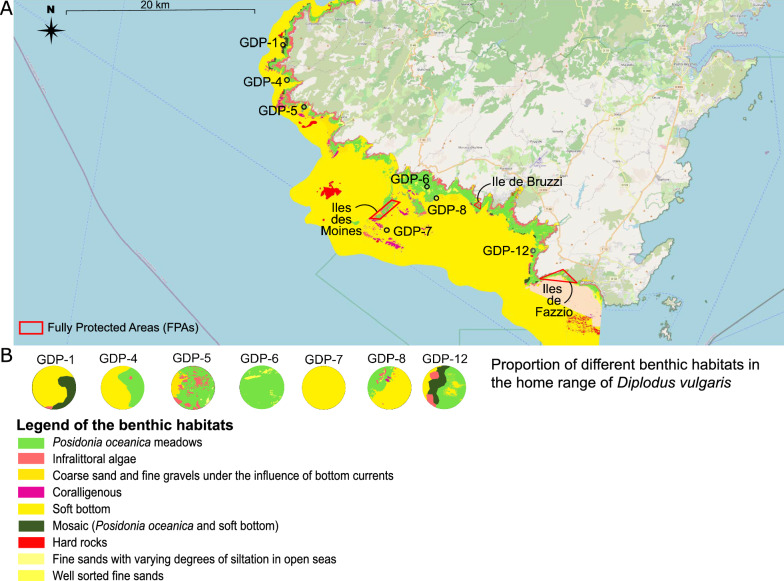
Benthic habitat types occurring in the study area. **A** Map of benthic habitats occurring in the BO region analyzed for the Small-scale dataset. This map was generated on the LOCUS platform (https://catalog-locus.univ-corse.fr/) using the publicly available CARTHAMED dataset (see Materials and methods; Equipe Ecosystèmes Littoraux, UMR CNRS SPE 6134 / FRES 3041, Università di Corsica, 2023. Cartographie continue des habitats marins en Corse - CARTHAMED - Secteur 5A. Consulté le 12/10/2023, https://catalog-locus.univ-corse.fr/layers/canope:carthamed:secteur_5a. Secteur 5B. Consulté le 12/10/2023, https://catalog-locus.univ-corse.fr/layers/canope:carthamed:secteur_5b. Secteur 6. Consulté le 12/10/2023, https://catalog-locus.univ-corse.fr/layers/canope:carthamed:secteur_6). **B** Profiles of the benthic habitats occurring in the home range of the *D. vulgaris* individuals collected in the different sampling locations

For the seabed habitat characterization, the publicly available CARTHAMED dataset (Equipe Ecosystèmes Littoraux, UMR CNRS SPE 6134/FRES 3041, Università di Corsica, 2023; [62]) was used. This dataset provides a continuous map of benthic habitats along the Corsican coast between 0 and 150 m of depth and it is publicly available at the LOCUS platform (https://catalog-locus.univ-corse.fr/.). Three sectors of this map were used to characterize the benthic habitat occurring in the environment exploited by the individuals of *D. vulgaris* (i.e. Sector 5A, Capo Pertusato—Roccapina; Sector 5B, Roccapine—Punta d’Eccica; Sector 6, Punta d’Eccica—Capo di Muru) (Fig. 2A).

To define the area of investigation around each sampling station regarding the type of benthic habitat(s), we used the average size of the total activity home range (the 95% of the Kernel Utilization Distribution, KUD) of *D. vulgaris* reported by [57], which is equal to 42,286.6 m^2^. The sampling station was considered as the center of the area and a radius of 232,08 m (i.e. defining an area twice larger than that of the 95% KUD reported in [57]) was chosen to set the edge of the home range. Eight different types of benthic habitats were recorded in the CARTHAMED dataset for our sampling stations: *Posidonia oceanica* meadows, infralittoral algae, coarse sand and fine gravels, soft bottom (e.g. detritic bottoms), coralligenous, mosaic (patches of *Posidonia oceanica* in soft bottom), well sorted fine sand and fine sand with varying degrees of siltation in open sea (Fig. 2B). In this study, the two last types of benthic habitat, both characterized by fine sand, were considered as a single category and classified as “Fine sand”. The proportion of the different benthic habitat types occurring in each circular area was measured using ImageJ [63] and expressed in percentages (Supplementary Metadata).

The distance between each sampling station and the centroids of the fully protected areas (FPAs) of îles des Moines, île de Bruzzi and îles de Fazzio (Fig. 2), was calculated by the *st_distance ()* function of the R package *sf* [64]*.*

### DNA extraction and 16S rRNA and COI amplicon gene sequencing

The bacterial DNA was isolated from ~ 350 mg of tissue obtained from the intestinal wall samples of 139 specimens. First, the intestinal segment was split longitudinally, and one of the two halves was used for the extraction of DNA. The diet DNA was isolated from ~ 250 mg of digesta previously homogenized by vortexing the sample at 2700 rpm for 2 min. To remove the RNAlater and the 70% ethanol, the intestinal wall and the digesta samples were centrifuged for 15 min at 1000g, rinsed with PBS 1× and centrifuged again for a further 15 min. Bacterial and diet DNA were isolated using the QIAMP Fast Stool Mini Prep kit (Cat. No. 51604, QIAGEN) following the protocol modifications reported in [31]. Negative controls were included in the DNA extraction procedure to check for cross and reagent contamination. The final DNA purity (A260/A280 and A260/A230) was measured with a UV–vis spectrophotometer Nanodrop (Thermofisher Scientific) and DNA concentration by fluorometry with Qubit (Thermofisher Scientific). DNA was stored at − 20°C until library preparation and sequencing.

The V4 region of the 16S rRNA marker gene for bacterial taxonomical identification was amplified and sequenced in three runs at StarSEQ GmbH (Mainz, Germany). The primers chosen for the amplification of the V4 region (~ 250 bp) were 515F (5′-GTGYCAGCMGCCGCGGTA-3′) and 806bR (5′-GGACTACNVGGGTWTCTAAT-3′) [65, 66]. Amplicons were sequenced on a MiSeq Illumina platform to generate paired-end reads.

For diet metabarcoding, library generation and amplicons sequencing were performed in one run at AllGenetics & Biology SL (http://www.allgenetics.eu; Coruña, Spain). A region of the mitochondrial COI gene of approximately 313 bp was amplified using the primers mlCOIintF (5′ GGWACWGGWTGAACWGTWTAYCCYCC 3′) and jgHCO2198 (5′ TAIACYTCIGGRTGICCRAARAAYCA 3′) [67, 68]. To avoid the amplification of the host *D. vulgaris* DNA, a blocking primer was designed by the company following the procedure reported in Vestheim & Jarman [69] using Geneious 11.1.5 (Biomatters Ltd). This blocking primer was Dvulgaris_mlCOIintF-BP (5′-ACCACTGGCAGGAAACCTTGCC-3′).

The PCR protocol used for the library preparation of both regions and information about the PCR positive and negative controls are reported in the Supplementary Materials and Methods..

### Processing of amplicon sequences

#### Processing of bacterial sequencing reads

The 16S rRNA gene sequence data obtained from the three sequencing runs were processed independently by the *dada2* pipeline [70]. Due to the drastically lower quality of the reverse reads, only the forward reads were eventually exploited (as explained in Dacey and Chain [71]). Reads were trimmed at 3′ for the length of the forward primer and at the first base with quality lower than Q = 30 at 5′. Reads displaying a number of expected errors higher than 2 and those assigned to the *PhiX* bacteriophage genome used as an internal standard during sequencing [72] were removed from the final dataset. After dereplicating, Amplicon Sequence Variants (ASVs) were inferred by the *dada2* algorithm [70] and chimeric sequences were removed. A decontaminated ASV table was obtained by removing all the ASV inferred from the reads sequenced in the negative controls. The three decontaminated ASV tables obtained separately for each sequencing run were merged together.

Taxonomical classification of the merged ASV table was performed using the Bayesian RDP classifier implemented in *dada2*, which relies on the SILVA rRNA gene database (release v138) [73]. Each ASV was assigned at the taxonomical level of kingdom, phylum, class, order, family and genus.

A few filtration steps were performed on the ASV table in order to remove all the ASVs belonging to mitochondria, chloroplasts and archaea and those occurring in only 1 sample. Additionally, samples with less than 8000 reads were also excluded from further analyses.

#### Processing of diet sequencing reads

The generation of an ASV table from the COI sequencing data followed the same steps just described for the 16S rRNA gene data. However, in this case both the forward and reverse reads were used and assembled to infer the diet profile of *D. vulgaris*.

ASVs were classified taxonomically by using both the BOLD [74] and NCBI nucleotide (nt) [75] databases as follows. First, the ASVs were classified by BOLDigger [76] that provided the taxonomical lineages and percentages of identity between the ASVs and the top 20 most similar sequences found in the BOLD database (i.e. hits). The taxonomical lineage assigned by BOLDigger to each hit was considered reliable depending on the percentage of identity displayed by the match: if higher or equal to 97%/95%/90%/85%/80% the classification was considered reliable down to the level of species/genus/family/order/class, respectively. All the hits with a percentage of identity lower than 80 were classified only at the phylum level. Then, the lineage occurring the most among the 20 hits and displaying the highest percentage of identity was selected as the final one for each ASV. The ASVs not classified by BOLDigger and those unclassified at the genus level by the steps just described, were reclassified by BLASTN using the NCBI nt database. A confidence threshold of 80% was set for the unclassified ASVs and of 95% for those classified down to genus level.

The ASVs represented by less than 3 reads in the dataset, those unclassified at the phylum level and those classified as *D. vulgaris* and *Homo sapiens* were removed from the final dataset.

### Diversity analyses

The bacterial ASVs associated to the samples included in the large-scale dataset (i.e. BA, CR, BO regions) and to those included in the small-scale dataset (only BO region) were analyzed separately. To ensure the robustness of the analyses, the bacterial community was not only explored at the ASV level, but also on bacterial data agglomerated at the level of genus, family, order, class and phylum. All statistical analyses were implemented in R studio (R Core Team, 2021).

#### Is geographic location a significant predictor of the diversity and composition of the gut microbiota?

The alpha-diversity of the bacterial communities was estimated by calculating the Shannon diversity index on the ASVs rarefied to 8000 reads per sample. The alpha-diversity of the bacterial communities was estimated by calculating the Shannon diversity index on the ASVs rarefied to 8000 reads per sample. The possibility that Shannon diversity index varies among the samples collected in the different sampling locations (both at a large and a small scale) was tested. For the samples included in the large scale dataset, a two-way ANOVA (or a two-way ANOVA on ranks, if the requirement of normal distribution and/or homogeneity of variance of the data were not met) was performed to test the effect of geographic location, sampling date and water temperature on the diversity of the gut microbiota of *D. vulgaris*. The two latter factors were included to rule out potential cofounding factors in case a significant effect of geographic location was observed. In contrast, because the samples included in the small scale dataset were collected over a short period of time and in one region, a one-way ANOVA tests (or Kruskal–Wallis tests, if the requirement of normal distribution and/or homogeneity of variance of the data were not met) was performed to determine only the effect of sampling location on the alpha diversity. In case of a significant outcome of the ANOVA tests, Tukey’s HSD test (or the non-parametric Dunn’s test) was performed as post-hoc test for pairwise comparisons between the geographic locations.

A compositional approach was applied to analyze the structure (i.e. composition) of the microbiota datasets as suggested by [77]: the zeros in the unrarefied ASV tables were replaced with near-zero counts by the cmultRepl() function of the R package “*zComposition*” [78] through the Bayesian multiplicative treatment [79]. Then, ASV tables were transformed by the centered log-ratio transformation (CLR) and pairwise dissimilarities were calculated among the fish individuals using the Aitchinson’s distance [80]. A Principal Component analysis (PCA) was implemented to explore the relationships between the samples based on their microbiota dissimilarities (beta-diversity).

The possibility that the dispersion of the dissimilarity values obtained would differ between the geographic locations (i.e. heterogeneity of variances) was tested by performing the Permutational Analysis of Multivariate Dispersion (PERMDISP) [81] through the *betadispers()* function from the *vegan* package in R [82]. The *vegan* function *adonis2()* was applied to define the effect of geographic location on the variation of the gut microbiota structure both at large and small scale. This function performs Permutational multivariate analysis of variance (PERMANOVA) if the covariate selected is a categorical one—as geographic location—; conversely, it performs linear regression with continuous covariates. In case of significant outcome of PERMANOVA, the *pairwise.adonis()* function from the pairwiseAdonis package in R [83] was applied to perform a multilevel pairwise comparison of the composition of the bacterial community in each geographic location.

Due to the significant differences observed in terms of total length of the fish both within the large-scale dataset and within the small-scale dataset, the relationship between this factor and the structure of the gut microbiota was further investigated in the two datasets. To do so, the total length of the fish was included in the *adonis2()* function together with the geographic location. Lastly, to exclude the potential cofounding effect of sampling date and water temperature on the composition dissimilarities between the individuals from the three regions (BA, CR and BO), these two factors were also included in the *adonis2()* function performed on the large scale dataset.

The bacterial genera differently abundant between the geographic locations were detected by ANCOM II on ASVs agglomerated at the genus level [84]. To confirm the results obtained by ANCOM II, the CLR abundances of the bacterial genera selected were also compared using one-way ANOVA and pairwise Tukey’s HSD test (or non-parametric Kruskal–Wallis test and non-parametric pairwise Dunn’s test).

The possibility that the composition of the core microbiota of *D. vulgaris* and of the non-core bacterial taxa in the community varied differently across the geographic locations was investigated in the large-scale dataset. First, the core microbiota was defined for each region separately (BA, CR and BO) as the bacterial genera occurring in at least 75% of the samples and with relative abundance higher than 0.01%. To detect these genera, the *core_members()* function of the R package *microbiome* [85] was performed on the rarefied ASVs agglomerated at the genus level. Then the PERMDISP test and PERMANOVA were applied to determine the relationship between the geographic location and the structure of the core microbiota. The same was done for the non-core bacterial community. The effect size (i.e. F values) and the significance (i.e. P values) provided by the PERMANOVA were used to define the amount of variance explained by the variable geographic location in each portion of the bacterial community (i.e. core and non-core).

#### The diet profile of *D. vulgaris* in BO

The diversity and structure of the diet of *D. vulgaris* was measured from the ASV table obtained for the COI mitochondrial region by the *dada2* processing and filtering.

The diet ASV table was rarefied down to the sample with the minimum number of reads (i.e. 7446 reads) and agglomerated to the different taxonomical levels (i.e. species, genus, family, order, class and phylum). The diversity of the diet was estimated by calculating the Shannon index on the rarefied datasets and the variation of this parameter across the sampling stations in BO was evaluated as described for the microbiota.

To generate the dissimilarity matrices needed to evaluate the differences in the composition of the diet in *D. vulgaris*, the same compositional approach performed on the bacterial data was applied on the diet profiles at all taxonomical levels. PERMANOVA was applied to the dissimilarity matrices to determine the contribution of the sampling location to the variation in the diet composition.

#### Which factors contribute to the variation of the gut microbiota across geographic locations at small scale?

Some factors that possibly contributed to the variation of both the alpha and beta diversity of *D. vulgaris* microbiota across the sampling stations in BO (small-scale dataset) were explored. These were: (i) the proportions of the seven types of benthic habitat in the area surrounding the sampling locations (see section "Characterization of the habitat features of the home ranges of *D. vulgaris* and measurement of distance from fully protected areas"), (ii) the distance of the sampling station from the closest Fully Protected Area (FPA) and (iii) the diversity and composition of the diet (i.e. number and identity of prey taxa found in the diet).

Distance based redundancy analysis (dbRDA) was implemented to determine the contribution of the the features characterizing the sampling station (i.e. proportions of the different benthic habitat types and distance from the FPA) to the variance of the microbiota composition dissimilarities. Six different dbRDA models were performed, each including a dissimilarity matrix obtained from the bacterial community agglomerated at different taxonomical levels (i.e. phylum, class, order, family, genus and non-agglomerated ASVs) as response variable. The collinearity among the explanatory variables was tested by the *vif.cca ()* function of the *vegan* package and the variables with a VIF (variance inflation factor) value higher than 20 were removed from the model [86]. Each model was tested by performing an ANOVA and in case of significance, a forward stepwise selection (*ordistep()* function from the R *vegan* package) was employed to select the variable contributing the most to the variance in the matrix. The percentage of variance explained by each final model was evaluated in terms of R^2^ adjusted and the contribution of each selected variable was defined by performing ANOVA.

Multiple Linear Regression (MLR) models were performed to investigate the relationship between the same features investigated in the dbRDA models and the alpha diversity of the bacterial community. Six different MLR models were performed each including as response variable the Shannon index calculated on the bacterial ASV table agglomerated at different taxonomical levels (i.e. phylum, class, order, family and genus) and non-agglomerated (i.e. at the ASV level). In order to identify the most important explanatory variables in each MLR model, we applied the best subset method with the *ols_step_best_subset ()* function of the *olrss ()* package in R [87]. All potential models were performed by fitting sequentially and independently all the explanatory variables; the Akaike information criterion (AIC), the R^2^ adjusted, and the Mallows’ Cp coefficient were estimated for each potential model; the model displaying the lowest AIC and Mallows’ Cp coefficient and the highest R^2^ adjusted was selected as the best one. The VIF (variance inflation factor) was measured for the variables included in the models selected by the best subset method to check for collinearity. In case collinear variables were detected, one of them was removed and the VIF recalculated.

Because the diet was analyzed only in a subset of samples included in the small-scale dataset, diet diversity and composition were not included as covariates neither in the dbRDA nor in the MLR models described above. Instead, the relationship between the Shannon diversity indexes of diet and gut microbiota was tested through Spearman’s correlation. To evaluate the contribution of diet diversity (i.e. Shannon index) to the dissimilarities between bacterial communities in terms of taxonomical composition (i.e. beta-diversity), the *adonis2()* function of the *vegan* package in R [82] was used. More specifically, a linear regression was fitted with the gut microbiota dissimilarity matrix as response matrix and the Shannon index of diet diversity as explanatory variable. Lastly, the association between the composition of the diet and that of the gut microbiota of *D. vulgaris* was investigated as follows. A Mantel test was performed between the dissimilarity matrix obtained for the diet and that obtained for the microbiota. As done previously, such as test were repeated at all taxonomical levels, both for the diet and for the microbiota.

### Inference of potential functions of the microbiota

The functional potential of the gut microbiota of *D. vulgaris* was predicted by the software PICRUST2 [88]. This analysis was performed independently in both the large-scale and the small-scale datasets.

The unrarefied and non-agglomerated bacterial ASV table was used as input for the PICRUST2 pipeline to generate a table of absolute abundances of gene families. First, PICRUST2 places the ASVs in a reference phylogeny to obtain the genetic profile of the phylogenetically closest genome for each ASV; then, the number of genes for each gene family found in the reference genomes and the total number of reads assigned to the ASV in the community are multiplied to predict the abundances of the gene families in the different samples. These abundances are eventually corrected for the number of 16S rRNA gene copies reported for each genome closely related to each ASV.

The gene families obtained by PICRUST2 were classified into Kyoto Encyclopedia of Genes (KEGG) orthologs (KOs) [89] and placed in broader KEGG metabolic pathways. We performed Hellinger’s transformation on the table of KEGG metabolic pathway abundances to correct for uneven sequencing of the samples, and 8 macro functional categories (each including several KEGG functional pathways) involved in the metabolism and homeostasis of the fish host were selected for the analyses. The KEGG functional pathways included in the analyses were selected based on previous reviews about the functionality of the fish gut microbiota [90]. These 8 macro-categories were: carbohydrate metabolism (15 KEGG pathways), amino acid metabolism (14 KEGG pathways), energy metabolism (8 KEGG pathways), lipid metabolism (16 KEGG pathways), glycan biosynthesis and metabolism (22 KEGG pathways), metabolism of cofactors and vitamins (12 KEGG pathways), metabolism of terpenoids and polyketides (21 KEGG pathways) and xenobiotics biodegradation and metabolism (21 KEGG pathways). The dissimilarities between samples regarding their functional potential were measured in terms of Bray–Curtis distances independently for each macro-category investigated. The contribution of the geographic location to the dissimilarities was explored using PERMANOVA (or Welch MANOVA if the condition of homogeneity of variances was not met by PERMDISP). Additionally, the metabolic pathways (KEGG pathways) enriched or underrepresented in the different geographic locations were detected by comparing their Hellinger transformed abundances using one-way ANOVA (or the non-parametric Kruskall-Wallis test).

In the small-scale dataset, simple linear regression was also calculated to test the relationship between the different features characterizing the sampling location in BO (i.e. proportions of different benthic habitat types and distance from FPA) and the functional potential of the microbiota using the *vegan* function *adonis2()*.

## Results

### Fish size

Fish from BA and BO had similar mean length (respectively 20.4 cm and 20.1 cm; Wilcox-Mann–Whitney test, P value = 0.89). They were significantly smaller than fish collected in CR (22.4 cm; Wilcoxon-Mann–Whitney test, P value = 0.0007 for the comparison of specimens from BA and CR, and P value = 0.01 for that of specimens from BO and CR). The mean size of the fish collected in BO for the small-scale dataset was equal to 19.9 cm and it also varied significantly across the sampling locations (ANOVA, P value = 0.001, F statistics = 4.14). The specimens from GDP-7 were the largest in the dataset and they significantly differed from the other specimens from BO (results of the pairwise Tukey’s test in Supplementary Fig. 1).

### Sequencing outcome

The three Illumina MiSeq sequencing runs of the 16S rRNA gene (V4 region) generated together 5,321,854 uncontaminated reads with an average number of reads per sample equal to 38,286. The *dada2* pipeline generated a total of 11,473 ASVs. Among these, 2311 ASVs were assigned to mitochondria and 122 ASVs to archaea and therefore excluded from further analyses. In addition, 80.7% of the ASVs (7297 ASVs) appeared only once in the dataset and were therefore discarded as well. The final dataset resulted in 1743 bacterial ASVs (Supplementary ASV Table). Finally, 9 specimens (1 from BO for the large-scale dataset and 8 from BO for the small-scale dataset) were discarded after filtering for a minimum of 8,000 reads per sample. The ASVs included in the final dataset belonged to 435 classified bacterial genera and to 92 unclassified genera.

The sequencing of the COI mitochondrial gene from 29 specimens included in the small-scale dataset generated 1,063,701 reads with an average of 35,456 reads per sample. A total of 1205 ASVs were obtained from the sequencing data processing by *dada2.* This amount was reduced after the filtration steps: 25 ASVs were represented by only 2 reads in the whole dataset, 7 ASVs were assigned to *D. vulgaris*, 2 ASVs to *Homo sapiens* and finally only 2 ASVs were not classified at the phylum level. Furthermore, 2 specimens were discarded after filtering for a minimum of 7,446 reads per sample. The final dataset contained 1169 ASVs, among which 35,5% were classified at the species level into a total of 175 different species*.*

### The taxonomical and functional composition of the *D. vulgaris* gut mucosal microbiota vary spatially at large scale

The Permutational Analysis of Multivariate Dispersion (PERMDISP) indicated no difference in the inter-individual variability of the gut mucosal microbiota composition for the three populations of *D. vulgaris* collected in the North-Western Mediterranean Sea (BO, CR, BA) (PERMDISP, P value > 0.05). Additionally, the gut mucosal microbiota structure was significantly different between the three populations (PERMANOVA at the ASV level, P value = 0.001; F statistics = 4.77). This was observed at all taxonomical levels (Supplementary Table 1). Conversely, neither the sampling date nor the water temperature seemed to contribute significantly to the composition dissimilarities observed: while no significant effect was found of the former factor at any taxonomical level, the latter was found to influence the structure of the microbiota only at the ASV level (Supplementary Table 1). Lastly, no relationship was found between the total length of fish specimens collected in the three regions and their gut microbiota composition (Supplementary Table 1).

**Fig. 3:**
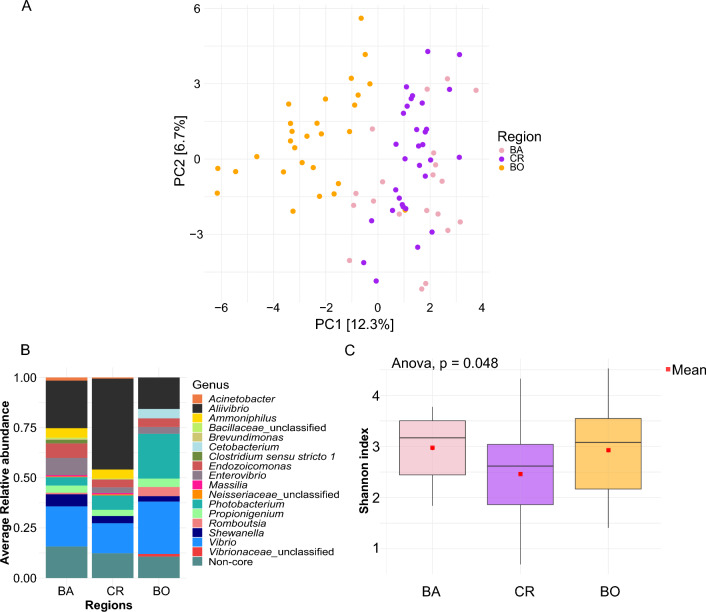
Gut microbiota structure and diversity of *D. vulgaris* investigated across a large geographic range in the NW Mediterranean Sea (Large-scale dataset). **A** Principal Component Analysis ordination representing the gut bacterial communities of specimens from the three geographic regions (BA, CR and BO). The distances were obtained by calculating the Aitchinson’s distances on the centered log-ratio (CLR) transformed abundances of the ASV (not agglomerated at higher taxonomical levels). **B** Stacked barplot representing the relative abundances of the core bacterial genera characterizing the gut microbiota of *D. vulgaris* in the NW Mediterranean Sea. Only the bacterial genera occurring in more than 75% of the individuals and representing more than 0.01% of their community were included in the core microbiota. **C** Boxplot of the Shannon alpha diversity index (at the ASV level) compared between the three different regions

By exploring the different composition of the gut microbiota at the ASV level through the PCA analysis (Fig. 3A), a separation between the specimens from BO and those from CR and BA samples was clearly observed. The pairwise statistical comparison of the beta dissimilarities revealed significant differences between all three locations (pairwise.adonis, P value = 0.01 for the comparison between BA and CR, P value = 0.003 for the comparison between BO and CR, and BA and BO). The contribution of the core and non-core bacterial genera to the microbiota dissimilarity observed across the three regions was further evaluated. The specimens collected in the three Mediterranean regions were characterized by a core gut microbiota represented only by 15 bacterial genera in BA, 12 in CR and 10 in BO (Fig. 3B; Supplementary Table 2). Although the number of core bacterial genera appeared relatively small compared to the number of bacterial genera detected in the *D.vulgaris* gut microbiota (527 in total), they were very abundant, as the number of reads assigned to these genera represented 84% of the total number of rarefied reads in BA, 87% in CR and 89% in BO. *Aliivibrio* and *Vibrio* were the most abundant genera in the fish gut microbiota from the three regions: together they represented 43% of the whole gut microbiota in BA, 60% in CR and the 41% in BO (Supplementary Table 2). Additionally, the core gut microbiota of the specimens from BO displayed a high abundance of the genus *Photobacterium* (22% of the whole gut microbiota). The genera *Brevundimonas* and *Clostridium *sensu stricto* 1* were only found in the core gut microbiota of the specimens from BA, while *Cetobacterium* and one unclassified genus of *Vibrionaceae* were only found in BO.

The differences observed in the structure of the core gut microbiota of *D. vulgaris* (i.e. beta diversity) were statistically confirmed by PERMANOVA (P value = 0.001, F statistics = 8.2251). Also, the structure of the non-core gut microbiota was observed to change between BA, CR and BO (P value = 0.001, F statistics = 2.89). The pairwise statistical comparison highlighted a significant dissimilarity of the core and non-core gut microbiota composition of BO compared to BA and CR (pairwise.adonis: P value = 0.003 obtained for both the core microbiota and the non-core microbiota). In contrast, only the composition of the non-core microbiota was different between the specimens from BA and those from CR (pairwise.adonis: P value = 0.012 obtained for the non-core microbiota; P value = 0.057 for the core microbiota).

Despite the differences observed in the microbiota composition, the overall diversity of the communities—Shannon index of alpha diversity—appeared to vary slightly across the three regions and only when calculated on the non-agglomerated ASVs (Fig. 3C; Supplementary Table 3) (two-way ANOVA P value = 0.04, F statistics = 3.37; pairwise Tukey's test with Bonferroni adjustment method, P value > 0.05 for all comparisons). Conversely, sampling date was the only factor that appeared to be significantly related to the variation of the diversity of the community at all taxonomical levels (two-way ANOVA, P value < 0.05 at all taxonomical levels; Supplementary Table 3). Overall, the mean value of Shannon index across the three regions was 2.99 ± 0.7.

The abundances of 19 bacterial genera were found to be different between the three fish populations by the ANCOM II test (Supplementary Fig. 2). The abundance of 18 of these bacterial genera significantly differed between BO and at least one of the other two populations. For example, BO displayed the highest abundances of the genera *Photobacterium*, *Paraclostridium*, *Cetobacterium* and the lowest for *Bacillus*, *Ammoniphilus*, *Brevundimonas*, *Massilia* and one unclassified genus included in the family of *Nesseiraceae* (Supplementary Fig. 2). By contrast, the BA and CR specimens differed by only 2 genera: *Romboutsia* and one unclassified genus from the *Mycoplasmataceae* family (Supplementary Fig. 2). Interestingly, of the 19 differently abundant bacterial genera, 8 were included in the core microbiota of the BA specimens, 6 in that of those from CR and only 4 in the BO ones (Supplementary Fig. 2).

**Fig. 4:**
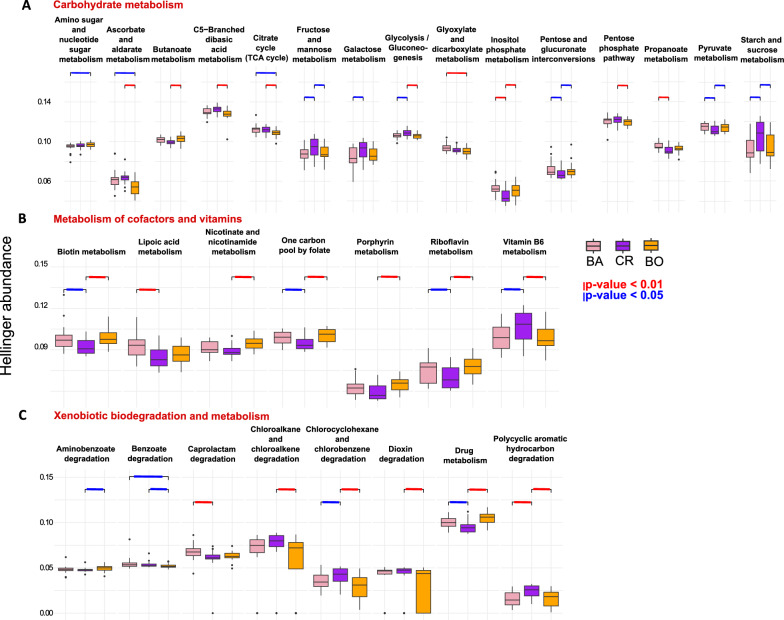
Functional prediction of the gut mucosa microbiota of *D. vulgaris* from the three regions. Boxplots representing the Hellinger transformed abundances of the KEGG metabolic pathways differently abundant in the gut microbiota of *D. vulgaris* from the three regions (BA in pink, CR in violet and BO in orange) according to the Kruskal–Wallis test. The metabolic pathways reported in this figure are included in the macro functional categories of: **A** carbohydrate metabolism; **B** metabolism of cofactors and vitamins; **C** xenobiotics metabolism and biodegradation. The P value of significant pairwise differences between regions (according to Dunn’s pairwise test) is reported over the boxplots

Functional profiling of the gut microbiota using PICRUST2 showed that 6 out of the 8 macro-categories of KEGG metabolic pathways involved in the metabolism and homeostasis of the fish, displayed a different composition according to geographic location, i.e. all except the energy metabolism category and the glycan biosynthesis and metabolism category (PERMANOVA, Supplementary Table 4). The gut microbiota of the specimens from the three regions appeared to be particularly different regarding the macro categories of carbohydrate metabolism and metabolism of cofactors and vitamins (see F statistics of PERMANOVA in Supplementary Table 4). This was especially true for CR (Supplementary Table 4). In the macro category of carbohydrate metabolism, the metabolic pathways of fructose, mannose, galactose, sucrose and starch were enriched in CR (Fig. 4A) while those of butanoate, pyruvate, propanoate and inositol phosphate were underrepresented in this population (Fig. 4A). In the macro-category of metabolism of cofactors and vitamins, all but one metabolic pathways looked underrepresented in the specimens from CR; the statistical test confirmed a significant difference with at least one of the two other regions for the metabolism of riboflavin, biotin and one carbon pool by folate (Fig. 4B). In addition, the specimens from CR harbored a distinct functional microbiota for the macro categories of amino acid and lipid metabolism (Supplementary Table 4).

In total, the relative abundance (Hellinger transformed) of 58 KEGG metabolic pathways was observed to vary significantly between the three populations (Supplementary Table 5; Fig. 4 and Supplementary Fig. 3). Overall, the metabolic pathway with the strongest significant difference between the three regions (Krustal-Wallis chi-squared value, Supplementary Table 5) was that classified as drugs metabolism in the macro-category of xenobiotics metabolism and biodegradation (Fig. 4C). The highest abundance was recorded in the region of BO, followed by that of BA and the lowest in that of CR. Other functions in the same macro-category showed differential abundances: the specimens from CR displayed the highest abundance of functions included in the metabolism and degradation of polycyclic aromatic hydrocarbons (PAH) and of chlorocyclohexane and chlorobenzene than those collected in the other two locations; similarly, those from BA were enriched for the functions included in the degradation of caprolactam.

### The gut microbiota of *D. vulgaris* vary taxonomically and functionally across a small spatial range

Similar to what was observed in the large-scale dataset, the taxonomic composition of the gut microbiota of the fish varied with sampling location at small spatial scale (only 33.6 km between the two most distant sampling locations). This result was obtained at all taxonomical levels except at the phylum level (PERMANOVA tests in Supplementary Table 1).

**Fig. 5:**
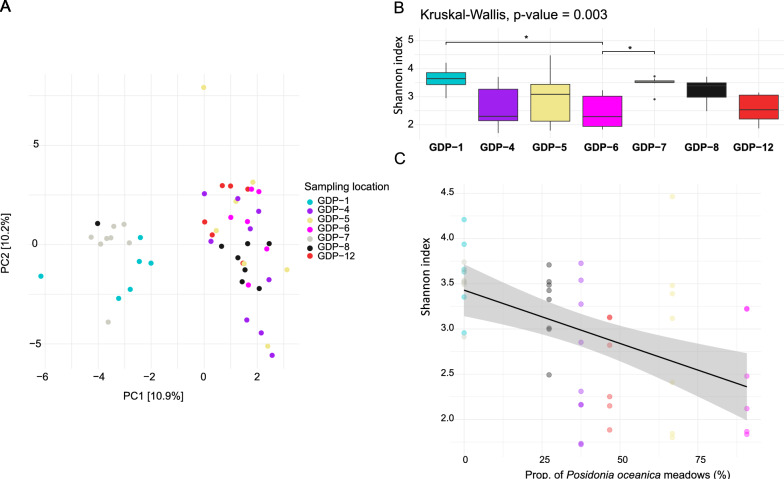
Gut microbiota structure and diversity of *D. vulgaris* investigated in BO (Small-scale dataset). **A** Principal Component Analysis ordination plot representing the gut bacterial communities of fish individuals from the seven sampling locations. The distances were obtained by calculating the Aitchinson’s distances on the CLR transformed abundances of the ASV (not agglomerated at higher taxonomical levels). **B** Shannon diversity index (at the ASV level) of the gut bacterial communities of individuals from the seven sampling locations. The P-value of significant pairwise differences between sampling locations (according to Dunn’s post hoc test) is reported over the boxplots (* P value < 0.05). **C** Linear regression calculated between the Shannon diversity index (at ASV level) and the proportion of the *Posidonia oceanica* meadows inferred in the home range of the individuals collected at each sampling location (Adjusted R^2^ = 0.22, P value = 0.0002). The shaded area represents a point-wise 95% confidence level

To define the pairs of sampling locations where the structure of the fish gut microbiota differed significantly, a pairwise post-hoc test (pairwise.adonis) was implemented. At the ASV level, 17 out of 21 pairwise comparisons between the sampling locations appeared to be significantly different (Supplementary Table 6). By repeating the pairwise analysis at all taxonomical resolutions, several pairs of sampling locations recursively displayed a significantly different bacterial community: GDP-8 and GDP-12, GDP-8 and GDP-4, GDP-1 and GDP-4, GDP-1 and GDP-12, and GDP-7 and GDP-4 (Supplementary Table 6). The specimens from GDP-1 and GDP-7 were observed to be clearly separated from the rest in the PCA ordination space (Fig. 5A). This separation resulted from different abundance of a few bacterial genera in the gut microbiota of the specimens from these two sampling locations and the rest of the samples (Supplementary Fig. 4). Indeed, of the 8 bacterial genera found to differ significantly across the sampling locations, two were overrepresent in GDP-1 and/or in GDP-7: *Moritella* in both locations (compared to all other sampling locations except GDP-6) and *Enterovibrio* in GDP-1 only (compared to GDP-5 and GDP-12) (Supplementary Fig. 4). Conversely, three bacterial genera were underrepresented in the gut microbiota of the fish in GDP-1 and/or in GDP-7: an unclassified genus included in the family *Vibrionaceae* in the specimens in both locations (compared to GDP-4, GDP-8 and GDP-12 for GDP-1, and to GDP-4 and GDP-8 for GDP-7), *Endozoicomonas* and one unclassified genus included in the family *Peptostreptococcaceae* in GDP-7 only (compared to GDP-4).

The alpha-diversity of the bacterial communities from the BO specimens appeared to vary accordingly with the sampling location when investigated at the ASV level (Fig. 5B). This was confirmed by extending the analysis at the taxonomical ranks of order, family and genus (Kruskal–Wallis, P value < 0.01). However, at the phylum and class levels, the gut bacterial communities were observed to be rather similar in terms of richness of the community (Kruskall-Wallis, P value = 0.09). The microbiota from the specimens collected in GDP-1 and GDP-7 displayed the highest Shannon index values and therefore the richest communities in the sample pool. However, the Shannon index from these two locations was significantly different from only one other location: GDP-6 (Dunn’s test: P value = 0.03 for the comparison between GDP-7 and GDP-6; P value = 0.04 for the comparison between GDP-1 and GDP-6).

The variation of the functional potential of the gut microbiota across a geographical range was investigated at small spatial scale by targeting the same 8 functional macro-categories of KEGG metabolic pathways targeted in the analysis at large spatial scale. Differently from what observed with the large-scale dataset, only the abundance of 2 out of the 8 macro-categories was found to vary significantly across the sampling locations: the metabolism of terpenoids and polyketides (PERMANOVA, P Value = 0.012, F value = 2.21) and the metabolism and biodegradation of xenobiotics (PERMANOVA, P value = 0.04, F value = 1.81). When comparing the KEGG metabolic pathways included in these two macro-categories, five pathways were found to be significantly different across the sampling locations: the biosyntesis of tetracycline (Kegg identifier: ko00253; P value = 0.02, Kruskall-Wallis chi-squared = 14.4) and of carotenoids (Kegg identifier: ko00906; P value = 0.01, Kruskall-Wallis chi-squared = 15.5), the degradation and metabolism of dioxins (Kegg identifier: ko00621; P value = 0.007, Kruskall-Wallis chi-squared = 17.5), of caprolactam (Kegg identifier: ko00930; P value = 0.004, Kruskall-Wallis chi-squared = 18.7) and of aminobenzoate (Kegg identifier: ko00627; P value = 0.01, Kruskall-Wallist chi-squared = 15.3). The pairwise comparison revealed that the specimens from GDP-1 displayed higher abundances of functions related to the biosynthesis of tetracycline (compared to those from GDP-12, GDP-6 and GDP-4) and of carotenoids (compared to GDP-6 and GDP-4) (Supplementary Table 7). Differently, the metabolism and degradation of aminobenzoate was underepresented in the specimens from GDP-1 when compared to GDP-8. Lastly, the metabolism of caprolactam was overepresented in the specimens from GDP-7 compared to those from GDP-4 and GDP-6 (Supplementary Table 7).

#### The type of benthic habitat and the distance from fully protected areas are predictors of gut mucosal microbiota features in *D. vulgaris*

Because the composition of the gut microbiota of *D. vulgaris* appeared to vary depending on the sampling location (except at the phylum level, Supplementary Table 1), we investigated the relationship between the dissimilarities of the gut microbiota composition and 9 features characterizing the sampling locations, i.e. the benthic habitat types and their distance from the FPAs. For that purpose, we performed 6 dbRDA (distance based redundancy analysis) models (i.e. one for each taxonomical level to which ASVs were agglomerated). Except the dbRDA model at the phylum level, all the other models confirmed the existence of this relationship (ANOVA, P value < 0.05 at all taxonomic resolution; P value = 0.24 at Phylum level; Supplementary Table 8). When the gut microbiota was investigated at the ASV level, 10.1% of the variation in its composition was predicted by the proportion of the *Posidonia oceanica* meadows, the distance from the closest fully protected area, the total number of different benthic habitats in the home range and the proportion of the coralligenous biocenosis (Supplementary Table 8). Among these, the most influential variable was the proportion of *Posidonia oceanica* meadows in the home range: indeed, this variable was a significant predictor for 4 out of the 5 dbRDA models (Table 1), followed by the proportion of coarse sand and fine gravels and the distance from the FPA (both variables significant in 3 out of 5 models, Table 1).

**Table 1:** Variables included in the 5 significant distance-based RDA models (tested at the ASV, genus, family, order and class levels) to evaluate the relationship between the structure of the gut microbiota of *D. vulgaris*, the benthic habitat and the distance from fully protected areas (FPAs)

Features characterizing the habitat	Number of times the variable was selected in the 5 dbRDA
Prop. of *Posidonia oceanica* meadows	4
Prop. of coarse and fine gravel under the influence of bottom currents	3
Distance from the closest FPA	3
Prop. of infralittoral algae	1
Number of benthic habitats	2
Prop. soft bottom	1
Prop coralligenous	1
Prop. of mosaic (*Posedonia oceanica*)	0
Proportion of fine sand	0

Further details on the models are reported in Supplementary Table 8. The total number of times each variable was finally selected in the models—after checking for collinearity among variables and executing a stepwise forward selection—is reported in the table

The relationship between the 9 features characterizing the sampling location and the alpha diversity of the gut microbiota was evaluated by performing six multiple linear regression (MLR) models (i.e. one for each taxonomical level to which the ASVs were agglomerated prior to calculations of Shannon indexes). The proportion of the *Posidonia oceanica* meadows, that of infralittoral algae and that of fine sand were the variables included in the MLR models selected by the best subset method at all taxonomical levels (Supplementary Table 9). Differently the distance of the sampling station from the FPA was never selected in the models. However, only the proportion of *Posidonia oceanica* meadows displayed a significant association with the diversity of the bacterial community whatever the taxonomical level (P value < 0.05 at all taxonomical levels; Supplementary Table 9). The negative coefficient reported for this variable in the output of the MLR models suggests the existence of an inverse relationship between the diversity of the bacterial community and the proportion of *Posidonia ocenainca* meadows in the benthic substratum (Fig. 5C).

Among the functional macro-categories observed to vary across the sampling stations in BO, only the metabolism of terpenoids and polyketides varied according to the proportion of the *Posidonia oceanica* meadows (linear regression, P value = 0.005, F statistics = 5.72) and of that of fine sand (linear regression, P value = 0.04, F statistics = 3.09).

#### No significant relationship is found between the diet and the gut mucosal microbiota taxonomical structure

The composition of prey families differed from one fish individual to another (Supplementary Fig. 5); however, at the phylum level the gut content was dominated by three taxa: Arthropoda (48% of the total abundance, occurring in the 100% of the samples), Annelida (28% of the total abundance, occurring in the 100% of the samples) and Mollusca (10% of the total abundance, occurring in the 96% of the samples).

Whatever the taxonomical level considered, the composition of the diet appeared to vary across the different sampling locations (PERMANOVA, P value < 0.05 at phylum level and P value < 0.01 at the class/order/family/genus/species levels). Despite both the composition of the diet and that of the gut microbiota were observed to differ depending on the sampling location, no direct relationship was found between them when investigated at all taxonomical resolutions: the Mantel test did not show any significant correlation between the dissimilarities found in the diets of the individuals and the composition of their gut microbiota (Mantel test, P value > 0.1 for the comparisons at all taxonomical levels). Similarly, the diversity of the diet (Shannon index) did not influence neither the structure of the gut microbiota (linear regression, P value > 0.1 at all taxonomic resolutions) nor its diversity (Spearman’s correlation, P value > 0.1 at all taxonomic resolutions).

## Discussion

### The gut mucosal microbiota of *D. vulgaris* is represented by a few core taxa and similar taxonomical diversity across a large geographical scale in the NW Mediterranean Sea

This study is the first to explore and to describe the gut microbiota of the two-banded sea bream (*D. vulgaris,* Geoffroy Saint-Hilaire 1817) by using a large and comprehensive dataset including 129 individuals from different geographical regions. Generally, when investigated through the analysis of the large scale dataset, the gut mucosal microbiota of the three populations displayed similar levels of Shannon diversity (Fig. 3C) and inter-individual variability of the bacterial community composition (Fig. 3A). Inter-individual variability is claimed to be often a consequence of stochastic changes in the microbiota that may occur in case of host’s stress or diseases [15]; increasing levels of inter-individual variability of the gut microbiota composition have been observed in populations of butterflyfish (*Chaetodon capistratus*) dwelling degraded coral reefs [19] and of sharpbelly (*Hemiculter leucisculus*) from polluted sites along the Ba river (China) [91]. Therefore, the similar inter-individual variability observed between the three populations investigated in this study may be interpreted as a sign of local stability and low levels of stress.

The core microbiota was explored to define the most commonly occurring and abundant bacterial genera characterizing this fish species. Seven core genera known to have a mutualistic relationship with marine fishes were identified in the gut microbiota of *D. vulgaris*: *Vibrio*, *Aliivibrio*, *Photobacterium*, *Enterovibrio*, *Endozoicomonas*, *Shewanella*, *Propionigenium* (Fig. 3B). *Vibrio* and *Aliivibrio* alone represented almost half of the whole gut bacterial community of the three populations. In samples from BO, these two genera and the genus *Photobacterium* represented 64% of the community. *Vibrio* is a very common genus in the intestine of marine fishes [2]. This includes many strains, and although some are harmful for fish, others have been described to confer protection against fish pathogens such as *Aeromonas salmonicida*, *Pasteurella piscicida* and *Listonella anguillarum* [2, 92]. Moreover, this genus contributes to the fish metabolism of several dietary compounds by producing amylase, lipase and chitinase [93, 94]. *Aliivibrio* correspond to the *Vibrio fischeri* group and it is phylogenetically and phenotypically distinct from other members of the *Vibrionaceae* family [95]. The genus *Aliivibrio* contains only six strains, four of which are consistently found in association with different marine vertebrates and invertebrates (*A. fischeri*, *A. logei*, *A. wodanis, A. sifiae and A. finisterris*) [96–99]. *Aliivibrio fischeri*, *A. sifiae* and *A. logei* have been described as bioluminescent [99], however the role of light-producing bacteria in the intestinal tract of fishes is not known yet. The genus *Photobacterium* was reported several times as a symbiont of carnivorous and omnivorous fishes, where it produces chitinases essential for the digestion of crustaceans in the diet [97].

In summary, our findings indicate that at the time of the investigation, *D. vulgaris* from the NW Mediterranean Sea held a stable gut bacterial community that was mainly composed of members of the family *Vibrionaceae*, especially *Vibrio* and *Aliivibrio*. Given the high abundance and occurrence of these bacterial genera, they can be considered resident of the gut microbiota of *D. vulgaris*. Therefore, future studies set in the NW-Mediterranean Sea pay attention to any major shift in the abundances, or the absence, of these bacterial genera in the gut mucosa microbiota of *D. vulgaris.*

Generally, the core microbiota is shaped by the selective pressure of the host intestinal system regardless of the environment [100]. However, to have a more complete overview of the core gut mucosal microbiota of *D. vulgaris* from the NW-Mediterranean Sea, the microbial data presented in this study should be coupled with data from other *D. vulgaris* populations, and with data from the same *D. vulgaris* populations at the reproductive season (from October to February). Indeed, during this season fishes undergo different environmental pressures due to their increased movement activity [57] and physiological alteration linked to the increased production of sex hormones that may influence the gut microbiota [101].

### The taxonomy and functionality of the gut mucosal microbiota of *D. vulgaris* is spatially heterogeneous in the Mediterranean Sea

#### Geographic location influences the taxonomy and potential functionality of the gut microbiota of *D. vulgaris* at a large geographic scale

At a large scale (i.e. the three regions BA, CR and BO), the taxonomical composition of the gut microbiota of *D. vulgaris* was observed to differ significantly at all taxonomic resolutions. Between the three populations, the one from BO showed the most unique bacterial community (Fig. 3A): 18 bacterial genera were differently abundant between the specimens from BO and those from at least one of the other two populations (Supplementary Fig. 2). Nevertheless, it is important to notice that most of these differently abundant genera were not representative of the bacterial community in BO as only 4 genera were included in the core microbiota of this population. Conversely, several genera reported to be differently abundant in BA and CR were recorded in the core microbiota of these two populations (Fig. 3B).

Analysis of the results obtained at large spatial scale can be useful to review the role that both the environment and the host’s genotype have on the development of the fish gut microbiota. The microbial colonization of the intestinal system of fish larvae occurs within the first 50 days of life. This microbiota colonizes the gut following the ingestion of suspended particles and egg debris by the fish larvae [41], from the surrounding water and from the first feed [2]. Therefore, the free-living bacterial taxa that are most abundant in the local environment and along the larval dispersal routes during this developmental stage may contribute to the final structure of the fish gut microbiota. Indeed, although the composition of seawater and sediment bacterial communities can vary greatly from those associated with fish gut, gills and skin, certain free-living bacterial taxa can benefit from the conditions of those body niches and establish within them [102]. In the Mediterranean Sea, an uneven distribution of environmental bacterial taxa (i.e. biogeography) was described in [103] and was shown to be a consequence of the interplay between different environmental parameters such as oxygen concentration and salinity, the longitude and the latitude of the sampling points. However, the composition of the free-living bacterial community is also influenced by the microbes specifically associated with the different marine macro-organisms (i.e. animals and plants): the contribution of macro-organisms in the dispersal and in the geographic distribution of marine microorganisms was indeed recently revealed [102]. Besides being the most distant region among the three, that of Corsica (BO) displays also higher salinity and water temperature compared to the Lion Gulf (BA and CR) (MARS3D model simulations, www.marc.ifremer.fr [60]). Temperature has been described to influence fish gut microbiota both in the wild and in controlled conditions [2, 104]. Temperature-related differences in microbiota composition are mainly due to the inability of fish to regulate their body temperature (i.e. ectothermic organisms) and the different temperature requirements of bacterial taxa. In this study, the different water temperatures reported for the sampling days in the three geographical regions by [60] contributed to explain the dissimilarities in the gut microbiota composition when those were analyzed at the ASV level. Beside displaying higher water temperature, BO is characterized by distinct biodiversity conditions both in terms of fish assemblage and coralligenous benthic species [105]. With this in mind, it is possible that the greater differences observed in the gut microbiota of the *D. vulgaris* from BO compared to BA and CR would be a consequence of the different environmental microbiota occurring in this region. In the future, the composition of the free-living bacterial communities living in the sediment and in the water column of the three regions investigated in this study should be further explored.

The contribution of the host genotype to the gut microbiota should also be considered further [7]. *D. vulgaris* is described to have a larval and juvenile dispersal range respectively, of 90 km and 165 km [106]. Given that the region of BO is more than 350 km away from CR and more than 450 km from BA, the possibility that the BO population is genetically separated from the other two is not unreasonable. However, this is currently unknown and therefore, a genetic analysis of the populations of *D. vulgaris* in the NW Mediterranean Sea will be required. In practical terms, this could be implemented by the combined analysis of mitochondrial DNA sequence markers and microsatellites to define the existing haplogroups for the three species in the Mediterranean Sea [107].

Despite the different microbiota structure found in the fishes from BO compared to those from BA and CR, the potential functionality of their microbiota was rather similar to that recorded for the fishes from BA. Conversely, the analysis of potential functions revealed that the bacterial communities from the CR region were significantly different from those from BA and BO for all the macro functional categories considered, except for the metabolism of terpenoids and polyketides, the energy metabolism, and the metabolism and biosynthesis of glycans (Supplementary Table 4).

The differences between the functional potential observed for the microbiota of the CR and BO populations are a consequence of their largely different microbiota taxonomic structure. Differently, those reported for the microbiota of the CR and BA populations—which were more similar taxonomically—may be caused by the differential abundance of a few functionally important bacterial taxa. The bacterial communities recorded in BA and CR were dissimilar only for the abundance of two bacterial genera: one unclassified genus of the *Mycoplasmataceae* family (more abundant in the samples from CR) and *Romboutsia (*more abundant in the samples from BA) (Supplementary Fig. 2). The genera included in the *Mycoplasmataceae* family are known for their important contribution to fish homeostasis and metabolism and among their several functions, they have been reported to support the metabolism of long chain polymers such as chitin and starch [18]. Indeed, the metabolism of starch was significantly more abundant in the samples from CR than those from BA (and BO) (Fig. 4A). Similarly, the genus *Rombustia* was described to be an important player in the metabolism of amino acids and of vitamins, except for vitamin B6 [108]. The higher abundance of this genus in the microbiota of individuals from BA, compared to those from CR, may explain why we inferred different abundances of genes linked to these metabolic pathways in the samples from the two regions (Fig. 4B).

Investigating the differential abundance of potential functions involved in metabolism and biodegradation of xenobiotics in the fish gut microbiota across a spatial range can be a starting point to detect compromised environments, as well as the distribution of specific pollutants [44, 109]. Even if CR is the most industrialized region among the three, being closely located to the area of Fos-Barre and to the commercial and touristic harbor of Marseille, a similar level of human impact was reported in the three regions (www.medtrix.fr in the IMPACT project [61]). In light of this, it was not surprising to find an overrepresentation of specific metabolic pathways linked to the degradation of xenobiotics in all three populations (Fig. 4C). Among all the metabolic pathways included in this category, the most discriminant one was that related to the degradation of drugs (i.e. pharmaceuticals), which was overrepresented in the gut microbiota of fish from the region of BO. Given this higher abundance in BO it would be worth investigating further the presence of these pollutants in the south western coast of Corsica. A recent study [110] set in the Western Mediterranean Sea reported that the highest concertations of the anti-inflammatory drug naproxen was detected in sites located around the Corsican island. Also the presence of seven other pharmaceuticals was detected at lower concentrations (diclofenac, ibuprofen, ketoprofen, paracetamol, caffeine, carbamazepine and sulfamethoxazole) [110]. Pharmaceuticals are an emerging source of pollution that reaches the sea through the river inputs as a main outflux of agricultural, urban and industrial runoff, through coastal wastewater treatment plants and through touristic coastal infrastructures; the distribution of pollution sources in the Mediterranean Sea needs a more intense monitoring [111]. To monitor the distribution of these pollutants in the Mediterranean Sea and more specifically its fauna, a possible approach would be to combine measurements of pollutant concentration in the fish tissues with the analysis of the fish gut microbiota for its biodegradation potential. Fishes such as *D. vulgaris* may be a good model for such an approach for two reasons: first, by being widespread in the Mediterranean Sea, they can be a good sentinel species; secondly, because they occupy a relatively high position in the trophic network (i.e. 3.5) [112] and therefore they are exposed to higher concentrations of pollutants through biomagnification [113].

#### Heterogeneity of the benthic habitats likely defines the taxonomy of the gut microbiota of *D. vulgaris* at a small geographic scale

Exploring the gut microbiota of 50 individuals in seven sampling locations distant from at most 33.6 km along the coast of the BO region allowed to define the effect of geographic location on the taxonomy and functionality of the microbiota at small spatial scale (small-scale dataset).

Surprisingly, regardless of the proximity of the sampling stations, the structure and the diversity of the bacterial communities associated with *D. vulgaris* varied according to the fish catching site (Fig. 5A, B). Although the extent of the variation was smaller than that observed at a large spatial scale, the statistical difference was observed at all taxonomical ranks except at the highest ones (i.e. phylum, class for the alpha diversity; phylum for beta diversity). The type of benthic habitat appeared to be the strongest determinant of the spatial variation of the gut microbiota. More specifically this was driven by the proportion of biocenosis of *Posidonia oceanica* meadows on the sea substratum (Table 1). In terrestrial animals, the type of habitat has been demonstrated to shape the structure of the gut microbiota in different classes. In the herbivorous howler monkeys (*Alouatta pigra*), the structure of the gut microbiota was observed to differ according to the type of vegetation present in the habitat (from evergreen to semi-deciduous forests) [50]. Similarly, blue tits (*Cyanistes caeruleus*) were found to harbor different gut microbiota communities depending whether they breed in dense deciduous forests or in meadows-like habitats [114]. In fishes, the gut microbiota structure was mostly described to change between freshwaters and marine habitat [39, 115]. However, in a recent multi-species study, the type of marine substratum (rocks, sand and detritic bottoms) was shown to be an important determinant of the diversity and structure of fish microbiota not only in the gut but also the skin and gills [42]. Both the bacterial communities living in the sediment and in the water column may influence the development of the gut microbiota in fishes [2]. In this regard, the presence of vegetation (i.e. seagrass) on the sea bottom has been shown to influence the composition of the sediment and free-living bacterial communities [116]: specifically, sediment and water samples collected in sampling sites characterized by unvegetated substratum displayed a distinct bacterial community composition compared to those collected in sampling sites with an increasing degree of vegetation (i.e. seagrass and algae, seagrass at low density, seagrass at high density). In this study, the sampling locations GDP-1 and GDP-7 were the only ones mostly surrounded by soft detritic bottom and completely lacking *Posidonia oceanica* meadows (Fig. 2). Thus, this difference in vegetation may have led to a distinct structure of the gut microbiota of the specimens collected in them (Fig. 5A), through a different composition of the bacterial communities living in the sediment and in the water column in this marine habitat.

The alpha-diversity of the fish gut microbiota was observed to be negatively related with the proportion of *Posidonia oceanica* meadows (Fig. 5C). This may suggest that the vegetated substrata are less favorable for this fish species’ gut microbiota, as higher alpha diversity is associated with better health and homeostasis in fishes [7]. Indeed, *D. vulgaris* is recorded frequently in rocky-algal and sandy substrata and only occasionally in *Posidonia oceanica* meadows [117].

The spatial difference in the composition of the gut microbiota observed at a small scale was also supported by the different distance of the sampling locations from the fully protected areas (i.e. FPAs) (Table 1, Supplementary Table 8). However, the alpha-diversity (Shannon index) of the microbiota did not correlate with the distance from the FPA. Although the fully protected areas are described to increase fish biomass and promote ecosystem restoration in the Mediterranean Sea and in the oceans [46, 118], omnivorous species are generally less affected by habitat protection—or degradation—compared to specialist ones [119]. Therefore, the result obtained for *D. vulgaris* in this study is in accordance with the generalist foraging strategy of this species. In fact, individuals living closer to the FPA would benefit from a more intact habitat and trophic network without major changes in the nutritional intake of their generalist diet. However, they would also experience higher inter-specific competition due to higher fish biomass. Conversely, those living further away would benefit from reduced inter-specific competition and still be able to find suitable foraging resources. In both scenarios, this adaptable species would emerge victorious, and it would not experience major changes in the alpha diversity of its gut microbiota.

Host’s diet is recognized to be one of the main predictors of the different gut microbiota composition; hence the relationship between the diet of *D. vulgaris*—inferred from the composition of the intestinal content—and the gut microbiota structure was investigated on a subset of the full small-scale dataset comprising 27 samples. Although the composition of the diet of *D. vulgaris* varied across the sampling locations (Supplementary Fig. 5), this appeared not to be a determinant of the structure and diversity of the mucosal gut microbiota of *D. vulgaris*. As previously stated, the autochthonous gut mucosal bacterial community is largely driven by the environment and the first feeds during the development of fish [2]; they lead at the adult stage to a few highly abundant bacterial genera representing almost entirely this community [120]. The latter represent a limit for the colonization and proliferation of external taxa that reach the intestine through the food and the water ingested. Therefore, while the type of resources exploited by the fish throughout its life time may influence the composition of the autochthonous gut mucosal microbiota, the gut content, which reflects the food ingested in the last few days, is less likely a determinant of this community. Differently, the gut lumen transient bacterial community is generally influenced by the short term diet as it is the one actively metabolizing the diet input [34]. Lastly, it is also important to consider again the generalist behavior of this species: analyzing the intestinal content after catching a fish provides only a snapshot of the diet, that might not be very representative of the general diet of the individual. In future studies, the relationship between the long term diet—obtained through the analysis of stable isotopes—and the structure of the gut mucosal community could be further investigated to determine their relationship in *D. vulgaris*.

Although the taxonomic composition of the gut microbiota was observed to differ across locations at a small scale, its functional potential was mainly conserved. Only five KEGG metabolic pathways were significantly different between the sampling locations, which reflected differences in the metabolism of terpenoids and polyketides and in the metabolism of xenobiotics. In the latter category, it was interesting to observe the higher abundance of the functions involved in the degradation of aminobenzoate in the samples collected in GDP-8 compared to those from GDP-1. 4-aminobenzoate (or *para*-aminobenzoic acid, or PABA) is a compound showing high UV absorption properties which makes it a common component of sunscreen products [121]. Its dispersion in the marine waters linked to seaside tourism makes it a harmful product for the marine environment [122]. Specifically, this compound is reported to have estrogen activity and to induce feminization in fish juveniles [123]. The higher potential ability to degrade aminobenzoate by the gut microbiota of specimens from GDP-8 might be linked to a higher concentration of aminobenzoate in the water surrounding this sampling station. According to the maps available on www.medtrix.fr in the IMPACT project [61, 124], the level of seaside tourism along the coast appears to be higher in front of the station GDP-8 than in front of the GDP-1 one (Supplementary Fig. 6). However, additional data on the concentration of 4-aminobenzoate in the area of this study are needed to confirm this speculation.

Although inferring the potential functions of *D. vulgaris* gut mucosal microbial community through PICRUST2 provided valuable insight into some specific metabolic pathways, it is important to stress the limits of such predictive methods [87]. Functional predictions are biased towards existing reference bacterial genomes, therefore the functions specific to an environment and performed by novel taxa are less likely to be identified. Secondly, the functions are predicted on short amplicon sequences (~ 250bp), therefore it is not possible to infer strain-specific functionality. In light of this, it is important that future studies investigate the functionality of the gut microbiota of *D. vulgaris* also through metagenomic and metatranscriptomic data: while the first would provide information about the potential functionality of the microbiota at the bacterial strain level, the second would inform on the functions effectively performed by the bacterial community.

## Conclusion

For the first time the gut mucosal microbiota of *D. vulgaris* was characterized by using a large dataset including specimens from three different geographic locations in the Mediterranean Sea. We report the genus *Aliivibrio*, *Vibrio* and *Photobacterium* as dominant taxa in the “natural” microbiota of *D. vulgaris*, at least for the populations living in the North-West Mediterranean Sea. Therefore, we suggest that future studies on the gut microbiota of *D. vulgaris* must treat with caution any major shift in the abundances of these three genera. We also reveal that the taxonomic and functional composition of the gut microbiota of *D. vulgaris* differ according to the geographic origin of the fish, to varying degrees depending on the spatial scale considered. At large scale, the Corsican population (BO) appeared to harbor the most distinct microbiota in terms of taxonomical composition. Conversely, one of the two population from the Lion Gulf (i.e. CR) displayed the most different set of potential functions. At small scale, we emphasize the role of the benthic habitat and the distance from fully protected areas as predictors of the spatial heterogeneity of the taxonomic composition of the gut microbiota. Specifically, we target the presence of *Posidonia oceanica* in the benthic habitat as predictor of both the microbiota composition and diversity. In contrast, we do not observe any contribution of the diet to the taxonomic composition of the gut microbiota of *D. vulgaris*.

In this study, we also suggest to consider the potential functionality of the gut microbiota of *D. vulgaris* to monitor pollutants in the Mediterranean Sea. We observed higher abundance of functions related to the drug metabolism in Corsica, where the levels of pharmaceuticals in the water have been reported to be higher than in other locations of the Mediterranean Sea. Moreover, we observed higher abundance of functions involved in the metabolism of aminobenzoate (component of sunscreen products) in specimens collected closely to sites undergoing seaside tourism in Corsica.

Lastly, given the important influence that the geographic origin has on both the taxonomical and functional features of the gut microbiota of *D. vulgaris*, we strongly advise future studies on this species to include specimens from multiple populations.

### Supplementary Information

Additional file1.

Fig. 1 Boxplots representing the total length of the *D. vulgaris* specimens across the seven sampling locations in BO. Only significant pairwise comparisons obtained using Tukey’s test are reported in the plot (**P value < 0.01).

Fig. 2 Boxplots representing the CLR transformed abundances of the gut bacterial genera indicated as differently abundant between the three regions (BA in pink, CR in violet and BO in orange) by ANCOM II and Kruskal–Wallis’ test. The P-value of significant pairwise differences between regions (according to Dunn’s post hoc test) is reported over the boxplots (* = P value < 0.05). The bacterial genera included in the core gut microbiota of *D. vulgaris* in each region are flagged with a star.

Fig. 3 Boxplots representing the Hellinger transformed abundances of the KEGG metabolic pathways differently abundant in the gut microbiota of *D. vulgaris* from the three regions (BA in pink, CR in violet and BO in orange) according to the Kruskal–Wallis test. The metabolic pathways reported in this figure are included in the macro functional categories of: A) lipid metabolism; B) amino acid metabolism; C) metabolism of terpenoids and polyketides. The P-value of significant pairwise differences between regions (according to Dunn’s post hoc test) is reported over the boxplots.

Fig. 4 Boxplots representing the CLR transformed abundances of the gut bacterial genera indicated as differently abundant between the seven sampling stations by ANCOM II and Kruskal–Wallis’ test. The P-value of significant pairwise differences between regions (according to Dunn’s post hoc test) is reported over the boxplots (* = P value < 0.05).

Fig. 5 Barplots representing the top 20 most abundant families of preys found in diet of *D. vulgaris* in BO. A portion of 313bp of the COI mitochondrial gene was used to obtain the diet profile of the individuals and the BOLD and NCBI (nt) databases were consulted for the taxonomical classification. Other prey families were included in “Other families”.

Fig. 6 Map of the seaside tourism pressure (“Tourisme balnéaire”) occurring in the BO region analyzed for the Small-scale dataset. Data from the IMPACT project publicly available at www.medtrix.fr were used to generate this map. Impact ranges between 0 (no touristic pressure) and 1 (intense touristic pressure). This was calculated in [61–124] through the support of data about touristic accommodations and second houses from the Institute National de la Statistique et Etudes Economiques (INSEE, www.insee.fr) and the locations of coastal beaches obtained from OpenStreet.

The PCR protocols performed to amplify both the V4 regions of the 16S rRNA gene and the 313 bp fragment of the COI gene prior amplicon sequencing with Illumina Miseq.

## Data Availability

The 16S rRNA gene sequence data generated and analyzed in this study have been deposited in the NCBI Sequences Read Archive (SRA, https://www.ncbi.nlm.nih.gov/sra) and are publicly available under the BioProject number PRJNA1081196. The COI gene sequence data are accessible at the same platform with the BioProject number PRJNA1081353. The SRA BioSample accession number generated for each sample are reported in the Supplementary Metadata. The codes used to perform the analyses are publicly available in the Github repository https://github.com/ginevralilli93/diplodusMed_micorbiota_spatialVariation.
