# Correction to: Do fish gut microbiotas vary across spatial scales? A case study of *Diplodus vulgaris* in the Mediterranean Sea

**DOI:** 10.1186/s42523-024-00333-4

**Published:** 2024-08-08

**Authors:** Ginevra Lilli, Charlotte Sirot, Hayley Campbell, Fanny Hermand, Deirdre Brophy, Jean‑François Flot, Conor T. Graham, Isabelle F. George

**Affiliations:** 1 Laboratoire d’Ecologie des Systèmes Aquatiques (ESA), Université Libre de Bruxelles (ULB), Brussels, 1050 Belgium; 2 Centre de Recherches Insulaires et Observatoire de l’Environnement (CRIOBE), University of Perpignan, Perpignan, France; 3 Marine and Freshwater Research Centre, Atlantic Technological University, Dublin Road, Galway, Ireland; 4 Evolutionary Biology and Ecology, Université libre de Bruxelles (ULB), Brussels, 1050 Belgium; 5 Interuniversity Institute of Bioinformatics in Brussels – (IB)², Brussels, 1050 Belgium

**Animal Microbiome (2024) 6:32**

10.1186/s42523-024-00319-2

Following publication of the original article [1], we have been notified that authors’ first and last names were switched.

They are now as follows:

Lilli Ginevra1*, Sirot Charlotte 2, Campbell Hayley 3, Hermand Fanny 1, Brophy Deirdre 3, Flot Jean-François 4,5

They should be as follows:

Ginevra Lilli 1*, Charlotte Sirot 2, Hayley Campbell 3, Fanny Hermand 1, Deirdre Brophy 3, Jean-François Flot 4,5
