# Supplementary figures and images for: Do fish gut microbiotas vary across spatial scales? A case study of Diplodus vulgaris in the Mediterranean Sea
Source: Anim Microbiome. 2024 Jun 13;6:32. doi: 10.1186/s42523-024-00319-2 (PMC11177387; doi:10.1186/s42523-024-00319-2)

Anova, F statistics = 4.14, P value = 0.001

Total length (cm)

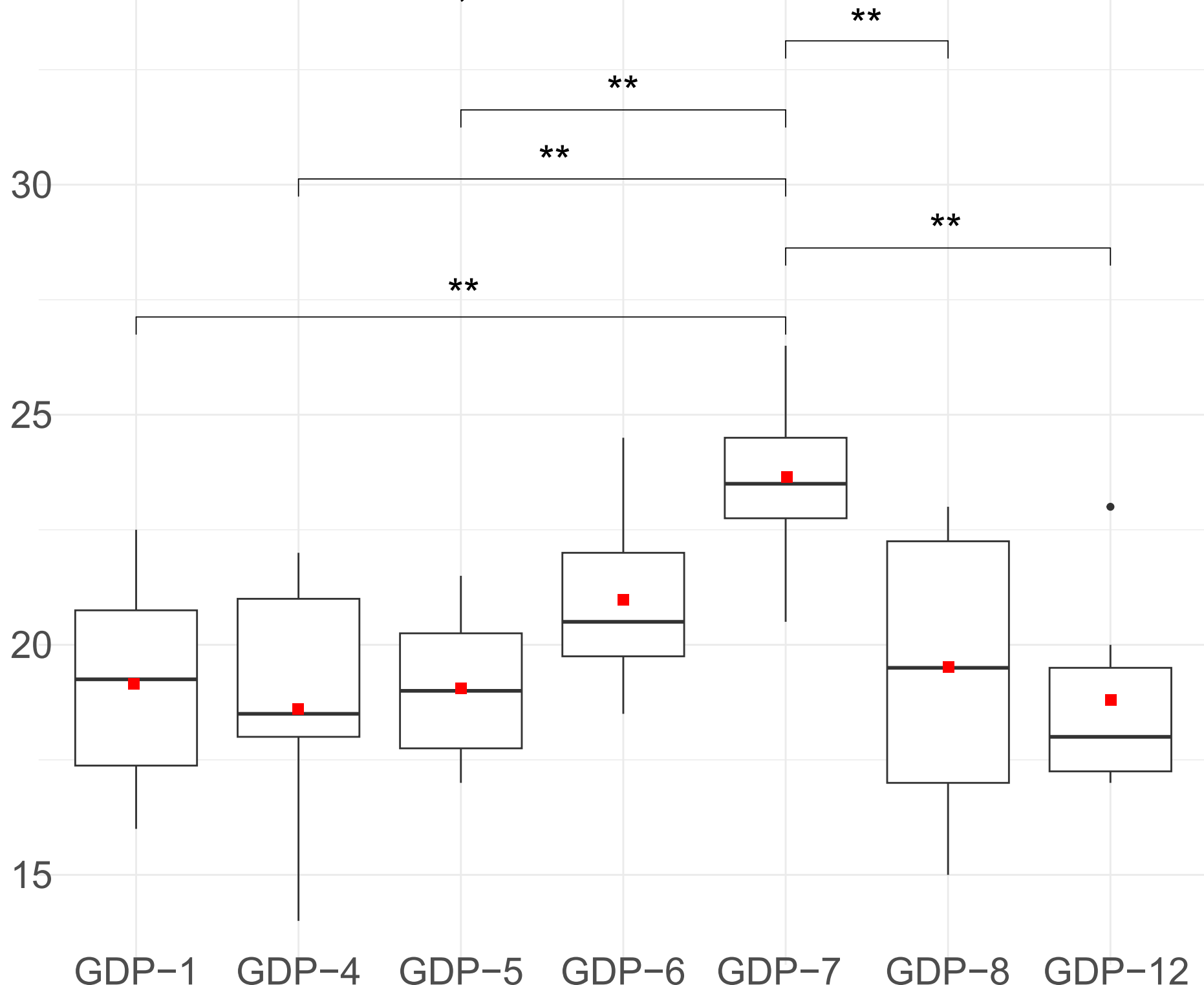

■ Mean

Supplement: Supplementary file 2 — Fig. 1 Boxplots representing the total length of the D. vulgaris specimens across the seven sampling locations in BO. Only significant pairwise comparisons obtained using Tukey’s test are reported in the plot (**P value < 0.01). [file 42523_2024_319_MOESM2_ESM.pdf]

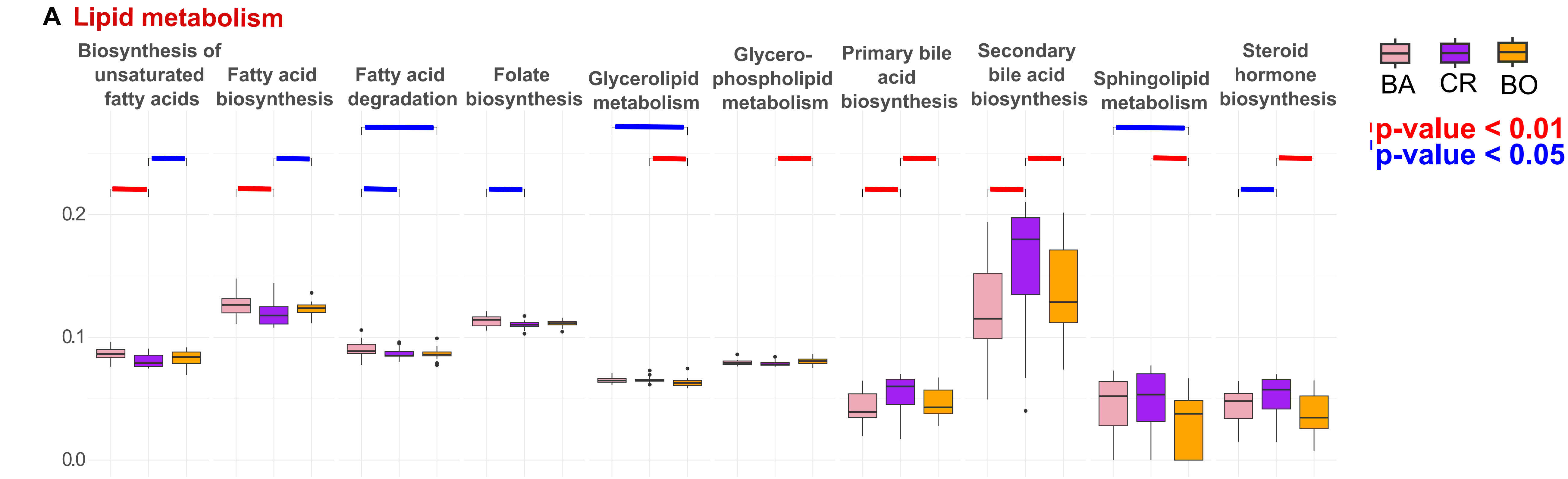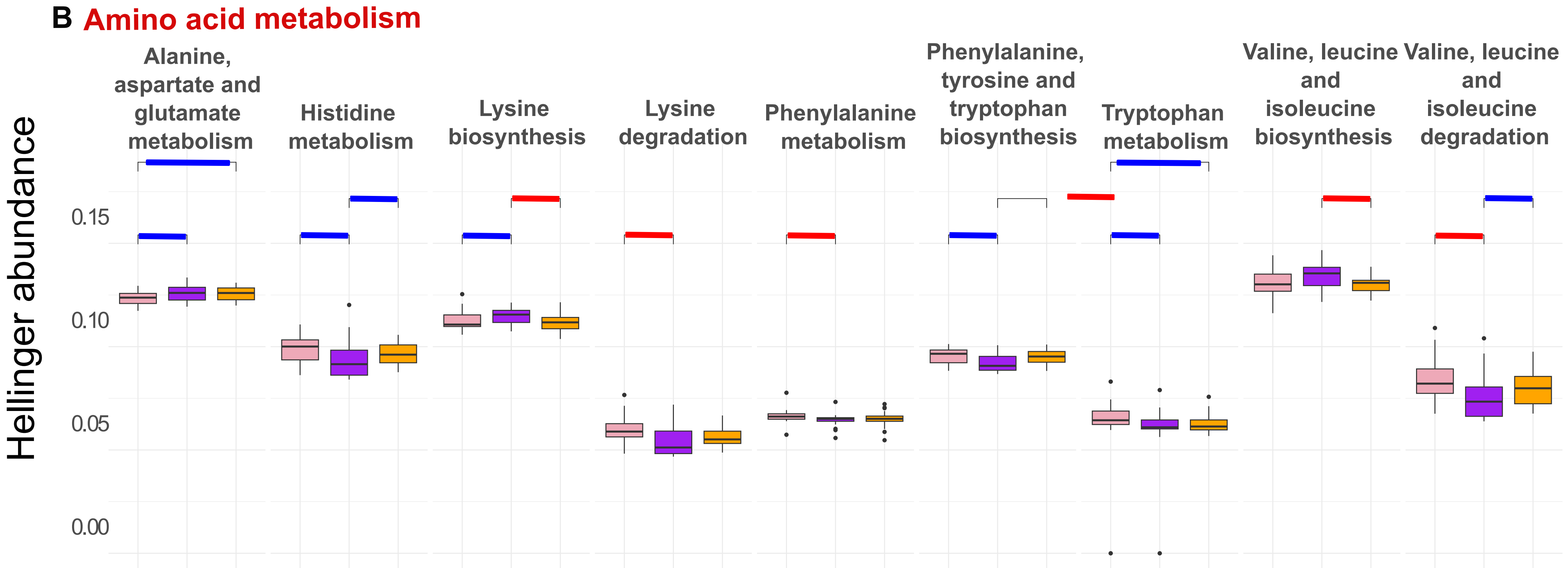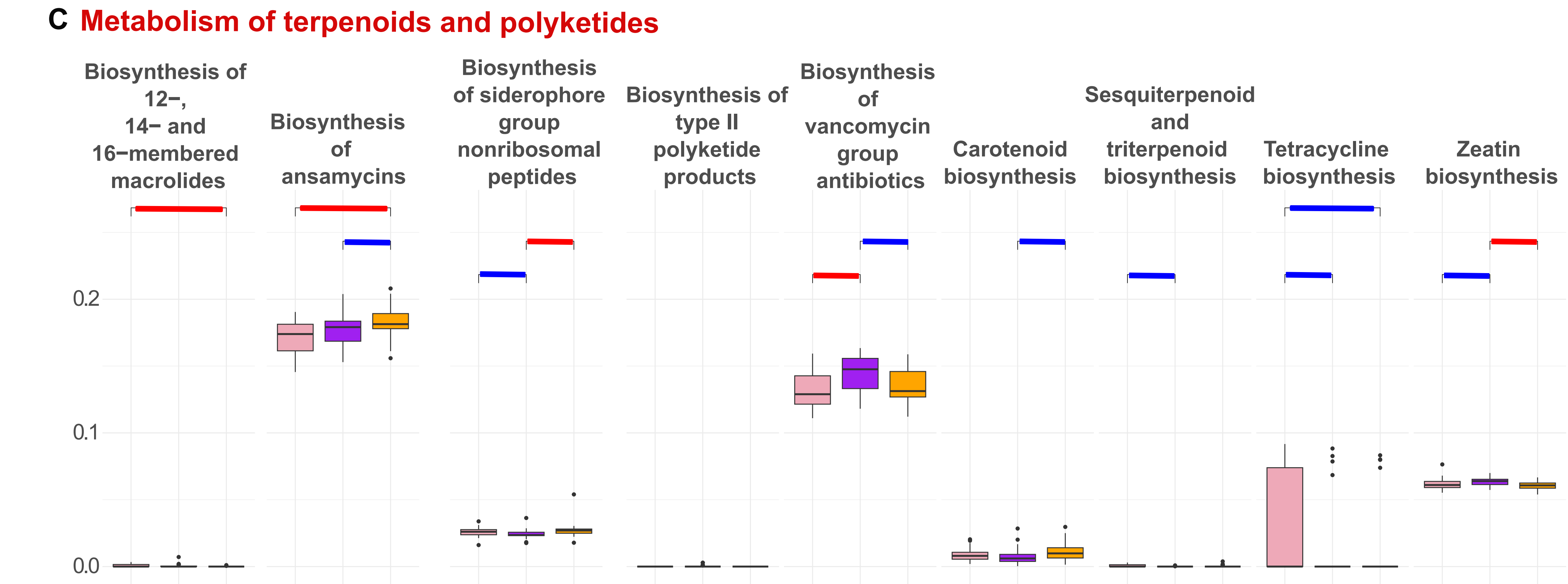

Supplement: Supplementary file 4 — Fig. 3 Boxplots representing the Hellinger transformed abundances of the KEGG metabolic pathways differently abundant in the gut microbiota of D. vulgaris from the three regions (BA in pink, CR in violet and BO in orange) according to the Kruskal–Wallis test. The metabolic pathways reported in this figure are included in the macro functional categories of: A) lipid metabolism; B) amino acid metabolism; C) metabolism of terpenoids and polyketides. The P-value of significant pairwise differences between regions (according to Dunn’s post hoc test) is reported over the boxplots. [file 42523_2024_319_MOESM4_ESM.pdf]

Relative abundance

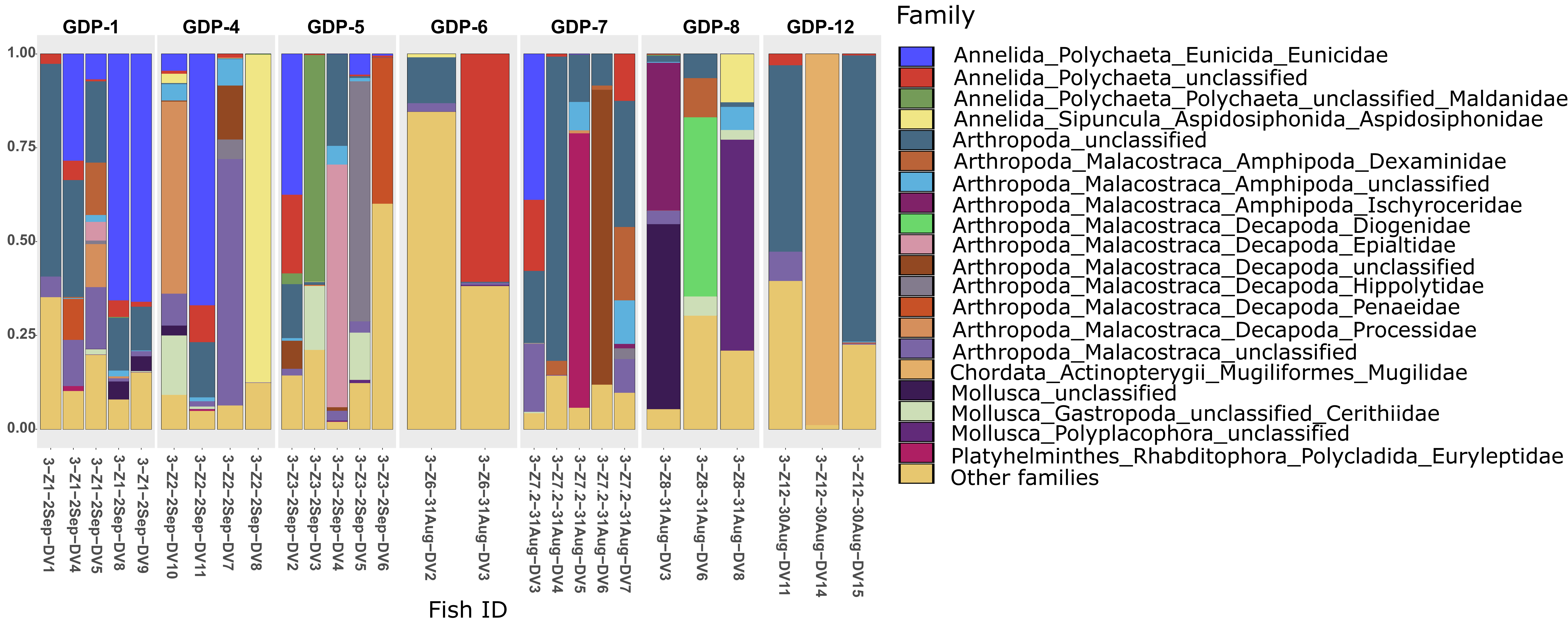

Supplement: Supplementary file 6 — Fig. 5 Barplots representing the top 20 most abundant families of preys found in diet of D. vulgaris in BO. A portion of 313bp of the COI mitochondrial gene was used to obtain the diet profile of the individuals and the BOLD and NCBI (nt) databases were consulted for the taxonomical classification. Other prey families were included in “Other families”. [file 42523_2024_319_MOESM6_ESM.pdf]

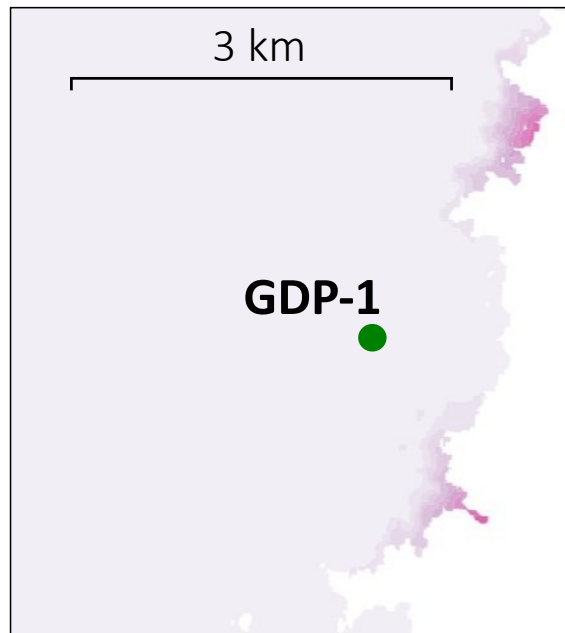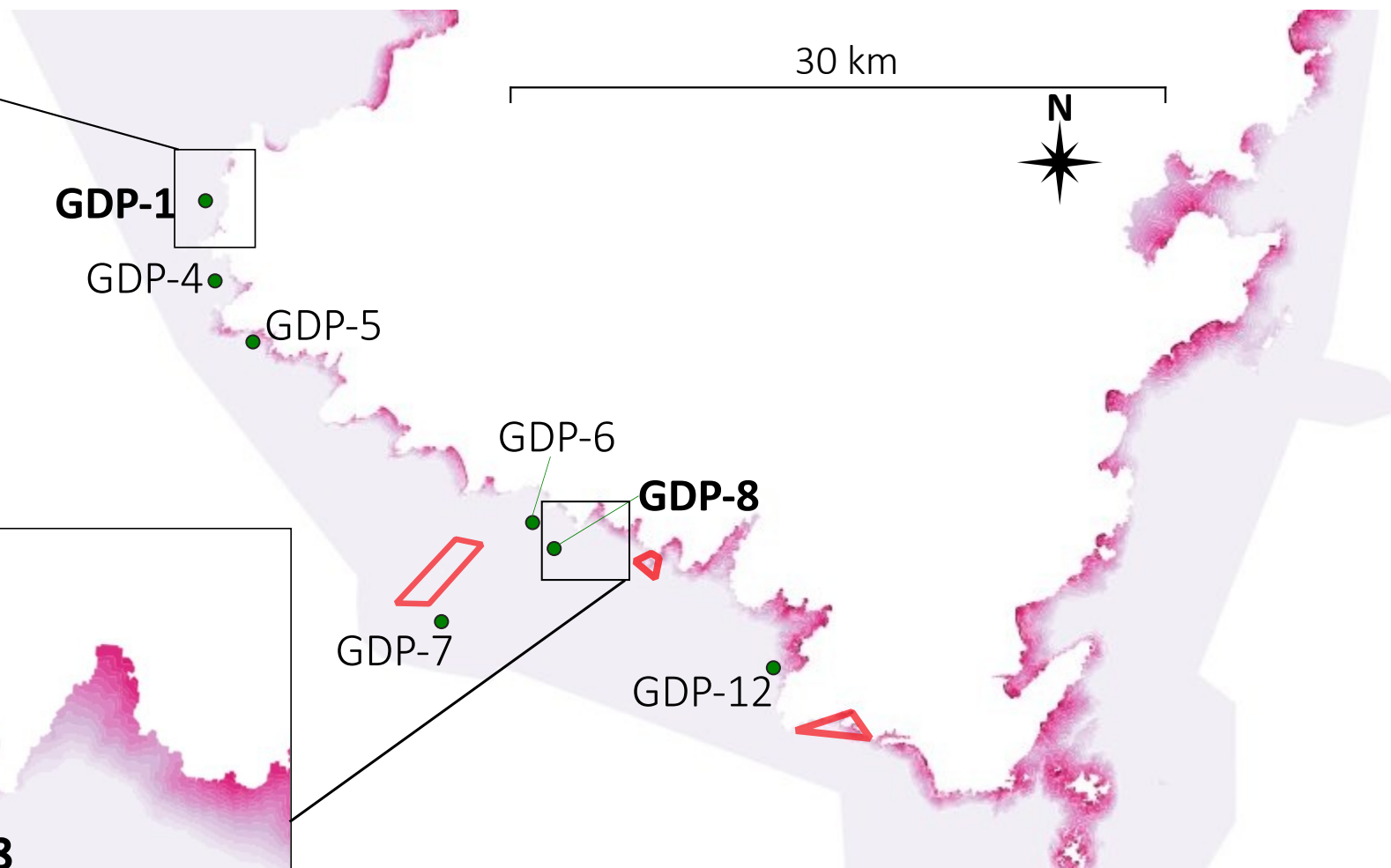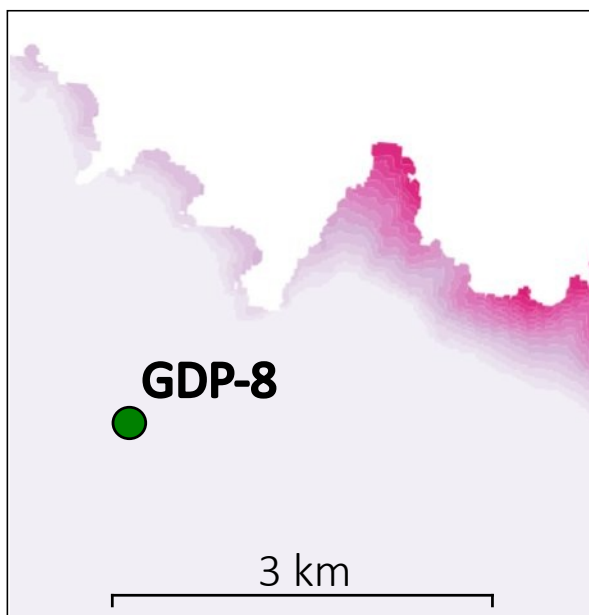

Impact level of seaside tourism

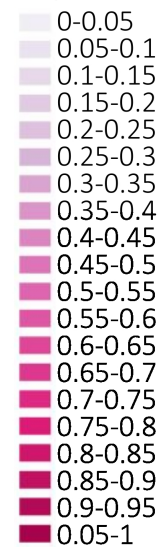

Supplement: Supplementary file 7 — Fig. 6 Map of the seaside tourism pressure (“Tourisme balnéaire”) occurring in the BO region analyzed for the Small-scale dataset. Data from the IMPACT project publicly available at www.medtrix.fr were used to generate this map. Impact ranges between 0 (no touristic pressure) and 1 (intense touristic pressure). This was calculated in [61–124] through the support of data about touristic accommodations and second houses from the Institute National de la Statistique et Etudes Economiques (INSEE, www.insee.fr) and the locations of coastal beaches obtained from OpenStreet. [file 42523_2024_319_MOESM7_ESM.pdf]
