# Supplementary material for: Do fish gut microbiotas vary across spatial scales? A case study of Diplodus vulgaris in the Mediterranean Sea
Source: Anim Microbiome. 2024 Jun 13;6:32. doi: 10.1186/s42523-024-00319-2 (PMC11177387; doi:10.1186/s42523-024-00319-2)
Supplement: Supplementary file 3 — Fig. 2 Boxplots representing the CLR transformed abundances of the gut bacterial genera indicated as differently abundant between the three regions (BA in pink, CR in violet and BO in orange) by ANCOM II and Kruskal–Wallis’ test. The P-value of significant pairwise differences between regions (according to Dunn’s post hoc test) is reported over the boxplots (* = P value < 0.05). The bacterial genera included in the core gut microbiota of D. vulgaris in each region are flagged with a star. [file 42523_2024_319_MOESM3_ESM.pdf]

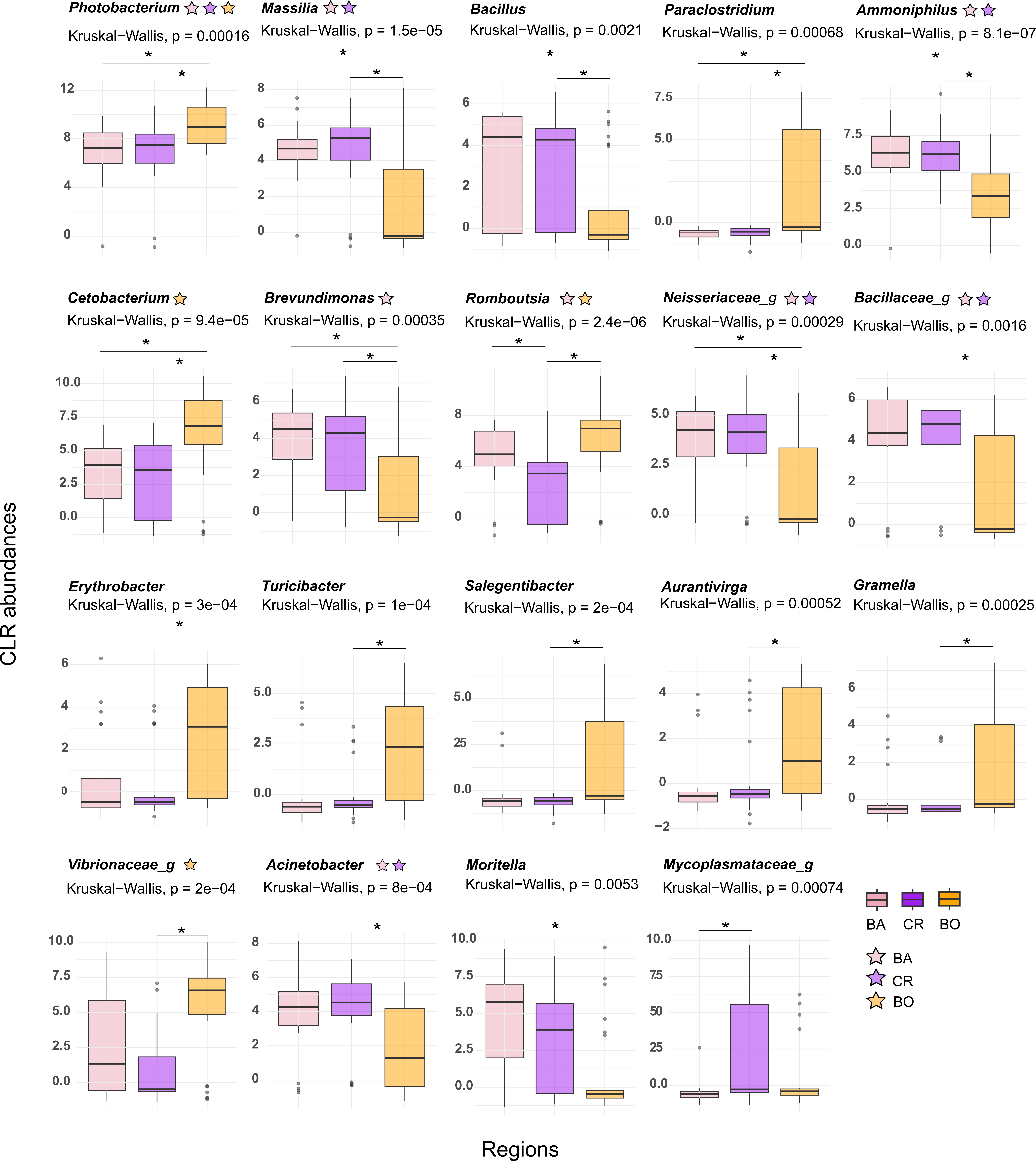

Photobacterium
Kruskal−Wallis, p = 0.00016
Massilia
Kruskal−Wallis, p = 1.5e−05
Bacillus
Kruskal−Wallis, p = 0.0021
Paraclostridium
Kruskal−Wallis, p = 0.00068
Ammoniphilus
Kruskal−Wallis, p = 8.1e−07
Cetobacterium
Kruskal−Wallis, p = 9.4e−05
Brevundimonas
Kruskal−Wallis, p = 0.00035
Romboutsia
Kruskal−Wallis, p = 2.4e−06
Neisseriaceae_g
Kruskal−Wallis, p = 0.00029
Bacillaceae_g
Kruskal−Wallis, p = 0.0016
Erythrobacter
Kruskal−Wallis, p = 3e−04
Turicibacter
Kruskal−Wallis, p = 1e−04
Salegentibacter
Kruskal−Wallis, p = 2e−04
Aurantivirga
Kruskal−Wallis, p = 0.00052
Gramella
Kruskal−Wallis, p = 0.00025
Vibrionaceae_g
Kruskal−Wallis, p = 2e−04
Acinetobacter
Kruskal−Wallis, p = 8e−04
Moritella
Kruskal−Wallis, p = 0.0053
Mycoplasmataceae_g
Kruskal−Wallis, p = 0.00074
BA CR BO
BA
CR
BO
CLR abundances
Regions
