# Supplementary material for: Do fish gut microbiotas vary across spatial scales? A case study of Diplodus vulgaris in the Mediterranean Sea
Source: Anim Microbiome. 2024 Jun 13;6:32. doi: 10.1186/s42523-024-00319-2 (PMC11177387; doi:10.1186/s42523-024-00319-2)
Supplement: Supplementary file 5 — Fig. 4 Boxplots representing the CLR transformed abundances of the gut bacterial genera indicated as differently abundant between the seven sampling stations by ANCOM II and Kruskal–Wallis’ test. The P-value of significant pairwise differences between regions (according to Dunn’s post hoc test) is reported over the boxplots (* = P value < 0.05). [file 42523_2024_319_MOESM5_ESM.pdf]

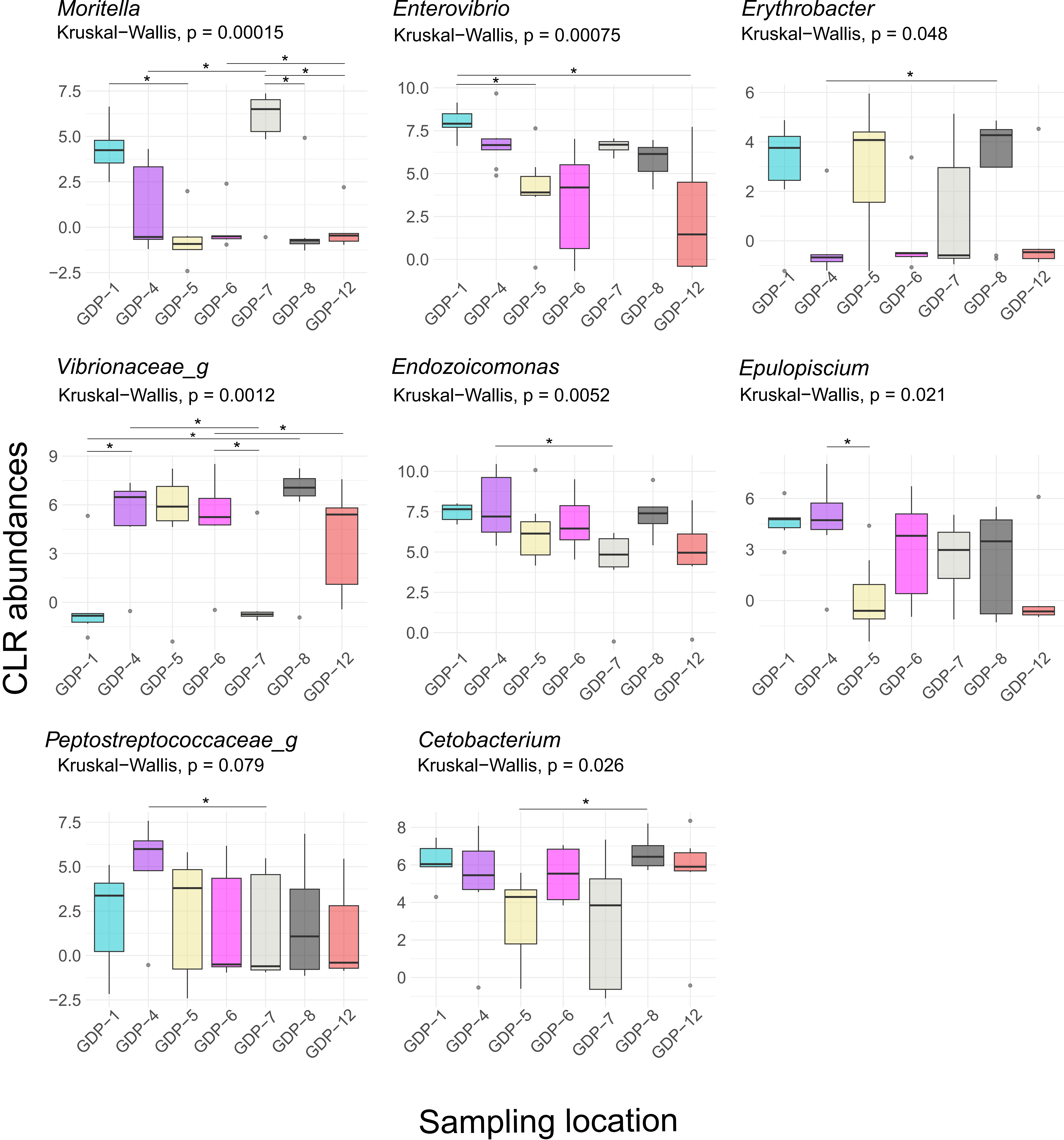
Moritella
Kruskal−Wallis, p = 0.00015
Enterovibrio
Kruskal−Wallis, p = 0.00075
Erythrobacter
Kruskal−Wallis, p = 0.048
Vibrionaceae_g
Kruskal−Wallis, p = 0.0012
Endozoicomonas
Kruskal−Wallis, p = 0.0052
Epulopiscium
Kruskal−Wallis, p = 0.021
Peptostreptococcaceae_g
Kruskal−Wallis, p = 0.079
Cetobacterium
Kruskal−Wallis, p = 0.026
GDP−1
GDP−4
GDP−5
GDP−6
GDP−7
GDP−8
GDP−12
CLR abundances
Sampling location
