# Supplementary material for: Do fish gut microbiotas vary across spatial scales? A case study of Diplodus vulgaris in the Mediterranean Sea
Source: Anim Microbiome. 2024 Jun 13;6:32. doi: 10.1186/s42523-024-00319-2 (PMC11177387; doi:10.1186/s42523-024-00319-2)
Supplement: Supplementary file 8 — The PCR protocols performed to amplify both the V4 regions of the 16S rRNA gene and the 313 bp fragment of the COI gene prior amplicon sequencing with Illumina Miseq. [file 42523_2024_319_MOESM8_ESM.docx]

**Supplementary Materials and Methods**

**PCR protocol performed by StarSEQ GmbH (Mainz, Germany) for the amplification of the V4 region of the 16S rRNA marker gene:**

Although the PCR conditions are a company secret adapted to their specific cyclers, StarSEQ GmbH has informed us that their protocol is inspired on that suggested for the Earth Microbiome Project ([https://earthmicrobiome.org/protocols-and-standards/16s/](https://eur01.safelinks.protection.outlook.com/?url=https%3A%2F%2Fearthmicrobiome.org%2Fprotocols-and-standards%2F16s%2F&data=05%7C02%7CLilli.Ginevra%40ulb.be%7C2a4b46ab04bd43796e6f08dc6a85d6f3%7C30a5145e75bd4212bb028ff9c0ea4ae9%7C0%7C0%7C638502370269517802%7CUnknown%7CTWFpbGZsb3d8eyJWIjoiMC4wLjAwMDAiLCJQIjoiV2luMzIiLCJBTiI6Ik1haWwiLCJXVCI6Mn0%3D%7C0%7C%7C%7C&sdata=FUTr5xPlsgp1LQkAK%2Fxh%2F3VKgbRcmmnpVnwZuU%2Bz90w%3D&reserved=0)) and that reported in [1] and [2].

They used a hotstart polymerase that does not have 3'-exonuclease activity and is free of any contaminating endo or exonuclease activities. It possesses 5'>3' DNA polymerase activity and a double-strand specific 5'>3' exonuclease.

**Thermocycler conditions of the Earth Microbiome Project protocol**

- Primers: 16S V4 515F–806R
- Amplicon size = 390 bp (including the primers, the Illumina indexes and the tags)

| **Temperature** | **Time, 96-well** | **Repeat** |
| --- | --- | --- |
| 94 °C | 3 min |  |
| 94 °C | 45 s | x35 |
| 50 °C | 60 s | x35 |
| 72 °C | 90 s | x35 |
| 72 °C | 10 min |  |
| 4 °C | hold |  |

34 cycles were run instead of the standard 35.

PCR negative and positive controls were included in the amplification and sequencing of the V4 region of the 16S rRNA marker gene. The negative control contained no DNA. The positive control consisted of a defined amount of DNA from different bacteria and fungi: ZymoBIOMICS™ Microbial Community DNA Standard (Catalog Nos. D6305 (200ng) and D6306 (2000ng), Zymo research Corp. The raw data generated for the negative and positive controls have been deposited at the NCBI Sequences Read Archive (SRA, <https://www.ncbi.nlm.nih.gov/sra>) and are publicly available under the BioProject number PRJNA954707 (BioProject generated for the study by Lilli et al., 2023 [3] including samples sequenced together with those used for the analyses of the current manuscript).

**PCR protocol performed by AllGenetics & Biology SL (http://www.allgenetics.eu; Coruña, Spain) for the library preparation of a fragment of the mitochondrial COI gene of 313 bp:**

The PCR amplification of this fragment was performed using the primers reported in the section 2.3 of the manuscript. To prevent the amplification of the host *Diplodus vulgaris* DNA a blocking primer was designed and used as described in the section 2.3 of the manuscript.

A C3 CPG spacer was added to the 3’ end of the blocking primer to prevent elongation.

The first amplification was performed using different dilutions made from the DNA extracts. PCRs were carried out in a final volume of 12.5 μL, containing 2.5 μL of template DNA, 0.5 μM of the primers, 10 μM of the blocking primers, 6.25 μL of Supreme NZYTaq 2x Green Master Mix (NZYTech), and CES 1X [4] The reaction mixture was incubated as follows: an initial denaturation step at 95 ºC for 5 min, followed by 35 cycles of 95 ºC for 30 s, 65 ºC for 45 s, 58.6 ºC for 45 s, 72 ºC for 45 s, and a final extension step at 72 ºC for 7 min.

Note: two primer annealing steps (65 ºC for 45 s and 58.6 ºC for 45 s) to enhance the binding of the blocking primer to the target DNA.

The oligonucleotide indices that are required for multiplexing different libraries in the same sequencing pool were attached in a second amplification step with identical conditions but only 5 cycles and 60 ºC as the annealing temperature.

A negative control that contained no DNA was included in every PCR round to check for contamination during library preparation. The raw data generated for the negative control have been deposited at the NCBI Sequences Read Archive (SRA, <https://www.ncbi.nlm.nih.gov/sra>) and are publicly available under the BioProject number PRJNA1081353, together with the rest of the raw diet data generated for the samples.

**References**

1. Caporaso, JG, Lauber, CL, Walters, WA, Berg-Lyons, D, Lozupone, CA, Turnbaugh, P. J., et al. Global patterns of 16S rRNA diversity at a depth of millions of sequences per sample. Proceedings of the national academy of sciences. 2011; 108 (supplement_1), 4516-4522.
2. Witzke, MC, Gullic, A, Yang, P, Bivens, NJ, Adkins, PRF, & Ericsson, AC. Influence of PCR cycle number on 16S rRNA gene amplicon sequencing of low biomass samples. Journal of microbiological methods. 2020; 176, 106033.
3. Lilli G, Sirot C, Campbell H, Brophy D, Graham C, George I. Geographic origin and host’s phylogeny are predictors of the gut mucosal microbiota diversity and composition in Mediterranean scorpionfishes (Scorpaena sp.). Front Mar Sci. 2023;1286706.
4. Ralser, M, Querfurth, R, Warnatz, HJ, Lehrach, H, Yaspo, ML, & Krobitsch, S. An efficient and economic enhancer mix for PCR. Biochemical and biophysical research communications. 2006; 347(3), 747–751. https://doi.org/10.1016/j.bbrc.2006.06.151
